# Mathematical modeling and control of lung cancer with *IL*_2_ cytokine and anti-PD-L1 inhibitor effects for low immune individuals

**DOI:** 10.1371/journal.pone.0299560

**Published:** 2024-03-14

**Authors:** Aqeel Ahmad, Muhammad Owais Kulachi, Muhammad Farman, Moin-ud-Din Junjua, Muhammad Bilal Riaz, Sidra Riaz

**Affiliations:** 1 Department of Mathematics, Ghazi University, D G Khan, Pakistan; 2 Department of Mathematics, Faculty of Arts and Sciences, Near East University, Northern Cyprus, Turkey; 3 Department of Computer Science and Mathematics, Lebanese American University, Beirut, Lebanon; 4 School of Mathematical Sciences, Zhejiang Normal University, Jinhua, Zhejiang, China; 5 IT4Innovations, VSB-Technical University of Ostrava, Ostrava, Czech Republic; 6 Mathematical Research Center, Faculty of Arts and Sciences, Near East University, Northern Cyprus, Turkey; Air University, PAKISTAN

## Abstract

Mathematical formulations are crucial in understanding the dynamics of disease spread within a community. The aim of this work is to examine that the Lung Cancer detection and treatment by introducing *IL*_2_ and anti-PD-L1 inhibitor for low immune individuals. Mathematical model is developed with the created hypothesis to increase immune system by antibody cell’s and Fractal-Fractional operator (FFO) is used to turn the model into a fractional order model. A newly developed system TCD*IL*_2_Z is examined both qualitatively and quantitatively in order to determine its stable position. The boundedness, positivity and uniqueness of the developed system are examined to ensure reliable bounded findings, which are essential properties of epidemic models. The global derivative is demonstrated to verify the positivity with linear growth and Lipschitz conditions are employed to identify the rate of effects in each sub-compartment. The system is investigated for global stability using Lyapunov first derivative functions to assess the overall impact of *IL*_2_ and anti-PD-L1 inhibitor for low immune individuals. Fractal fractional operator is used to derive reliable solution using Mittag-Leffler kernel. In fractal-fractional operators, fractal represents the dimensions of the spread of the disease and fractional represents the fractional ordered derivative operator. We use combine operators to see real behavior of spread as well as control of lung cancer with different dimensions and continuous monitoring. Simulations are conducted to observe the symptomatic and asymptomatic effects of Lung Cancer disease to verify the relationship of *IL*_2_, anti-PD-L1 inhibitor and immune system. Also identify the real situation of the control for lung cancer disease after detection and treatment by introducing *IL*_2_ cytokine and anti-PD-L1 inhibitor which helps to generate anti-cancer cells of the patients. Such type of investigation will be useful to investigate the spread of disease as well as helpful in developing control strategies from our justified outcomes.

## 1 Introduction

Mathematics first found its application in biology during the 13^*th*^ century when Fibonacci introduced the renowned Fibonacci series to explain population growth. Daniel Bernoulli later employed mathematical principles to describe the effects on small organisms’ shapes, while in 1901, Johannes Reinke coined the term “bio math”. Bio math is essentially the theoretical examination of mathematical models to analyze the underlying principles governing the structure and behavior of biological systems.

Over the past few decades, there has been a remarkable surge in biological sciences, and it is reasonable to expect that this trend will persist, driven by significant technological advancements. Society has consistently derived benefits from and made substantial contributions to mathematics. Mathematics has played a pivotal role in advancing the natural sciences and can similarly revolutionize biological research [1]. Mathematics provides us with models to help us understand the intriguing complexities posed by biology, and biology, in turn, assesses these mathematical models. Complex mathematical problems are now easier to handle thanks to recent advances in computer algebra systems. Consequently, this frees up researchers to concentrate on understanding mathematical biology instead of figuring out how to solve problems [2].

Cancer is a highly complex subject, encompassing a wide array of diseases with unique characteristics, numbering at around two hundred. Consequently, numerous researchers persist in their efforts to investigate the interactions between immune cells and tumor cells. They employ diverse methodologies to gain deeper insights into the dynamics of cancer [3, 4]. The primary focus of this research lies in understanding the interplay between the immune system and tumor cells [5]. The application of ordinary differential equations in mathematical modeling has proven to be indispensable in comprehending the dynamics of the tumor-immune system. This sheds light on how host immune cells and cancer cells interact and develop [6–8]. Nevertheless, it’s worth noting that fractional-order differential equations offer additional features when compared to traditional derivatives in mathematical modeling.

Fractional calculus has gained significant recognition and importance due to its established usefulness in various scientific fields, including systems biology [9] and other branches of science [10]. Fractional calculus enables the application of derivatives and integrals of non-integer orders. An advantage of fractional derivatives, as well as integrals, lies in their non-local characteristics [11]. In the field of epidemiology, mathematical models are frequently utilized to gain a better understanding of the intricacies of infectious diseases. Stability theory for differential equations is employed to analyze the modeling approach for dysentery with controls [12]. The commonly utilized operators, such as Caputo-Fabrizio and Atangana-Baleanu, incorporate local derivatives along with exponential functions, power laws, and Mittag-Leffler functions, respectively.

Cancer is a significant global health concern, surpassing AIDS, tuberculosis, and malaria combined in terms of its impact on human lives. It affects one in every six individuals worldwide. At present, it is the second most common cause of mortality worldwide, and is more common in countries with high or very high Human Development Index (HDI) [13]. The incidence of new cancer cases and fatalities continues to increase, owing to factors like rising life expectancy and shifts in epidemiology and demographics. By 2030, Sustainable Development Goal (SDG) 3.4 strives for a 33% reduction in premature mortality linked to noncommunicable diseases (NCDs), which encompass cancer. Unfortunately, progress in the field of cancer research and treatment has lagged behind other NCDs. In 2018 alone, there were an estimated 18.1 million newly diagnosed cases of cancer and 9.6 million cancer-related deaths. Regionally, the incidence rates were distributed as follows: 48.4% in Asia, 21.0% in the Americas, 23.4% in Europe, 5.8% in Africa, and 1.4% in Oceania [14].

Lung cancer stands out as a prominent contributor to cancer-related deaths, accounting for roughly a quarter of all cancer fatalities and surpassing the combined mortality of colon, breast, and prostate cancers [15]. In 2018, nations with both high and medium Human Development Index (HDI) levels reported exceptionally high rates of lung cancer diagnosis among men [13]. Notably, Bangladesh recorded a prevalence of 13.1% among men and 2% among women over the preceding 5 years [16]. The World Health Organization (WHO) data from 2018 highlights a concerning scenario in Bangladesh, with 108,137 cancer-related deaths and 150,781 cancer cases. Lung cancer constituted 8.2% of these cases, with an associated fatality rate of 11% [17]. The situation is expected to worsen over time, as WHO predicts an increase in lung cancer cases from 10,851 in 2012 to 12,374 in 2018, and a projected 26,738 cases by 2040. Thus, by 2040, lung cancer is expected to become a greater concern than breast cancer. Several investigations have examined the dynamics of cancer. A mathematical model for studying the genesis of cancer was presented by De Pillis et al. [18]. To treat cancer patients, they used a combination of chemotherapy, activation protein injections, TIL injections, and IL-2 injections. Furthermore, De Pillis et al. [19] presented a unique mathematical model that clarifies the relationship between tumors and the immune system, emphasizing the functions of CD8+ T cells and natural killer (NK) cells in immune-mediated tumor rejection. Another mathematical model utilizing dendritic cells for patient therapy was created by Trisilowati et al. [20]. Natural killer cells were substituted in their model for cytotoxic T lymphocytes and dendritic cells as the main immunological components. In reaction to these advancements, Unni and Seshaiyer [21] developed a mathematical model that explains how tumor cells interact with different immune cells, such as dendritic cells, CD8+ T cells, and natural killer cells. The distribution of drugs to certain cell sites is also covered by this approach.

Research by Kirschner and Tsygvintsev [22] describes how they developed a novel tumor treatment technique. Additionally, they created a mathematical model to forecast how the immune system will react to cancers. By using host components, their therapy strategy seeks to improve the immune response. Expanding on these findings, Kirschner and Panetta used mathematical modeling to clarify the relationships among IL-2, immune-effector cells, and tumor cells [23]. Both long-term tumor recurrence and transient variations in tumor development may be addressed by these research endeavors. Subsequently, the scientists conducted experiments on mice to examine the effects of adoptive cellular immunotherapy and identified the specific conditions conducive to tumor eradication. Decker and colleagues (Decker et al., [24]) undertook a thorough study of the literature to pinpoint and outline significant turning points in the acceptance of immunotherapy as an effective treatment for neoplastic cancers. The development of crucial model systems, notably in mouse and dog models, was another area of emphasis in their research.

In a related study, Waldman and his colleagues (Waldman et al., [25]) offered an extensive historical and biological perspective on the development and clinical utilization of cancer immunotherapy. They highlighted the crucial involvement of T lymphocyte regulation and examined a variety of clinical trials that demonstrated the efficacy of distinct drug categories. They also discussed the associated adverse effects. Furthermore, Hiam-Galvez and their team (Hiam-Galvez et al., [26]) furnished a summary of the contemporary insights regarding systemic immunity in cancer. Their research encapsulated the current knowledge regarding this vital aspect of cancer research.

Kartono [27] developed a mathematical model to illustrate the impact of tumor-infiltrating lymphocytes (TIL), interleukin-2 (IL-2), and interferon-alpha on tumor cell behavior. McLane et al. [28] provided a comprehensive overview of Tex cells, encompassing their developmental pathways, transcriptional and epigenetic characteristics, as well as intrinsic and extrinsic factors contributing to cell exhaustion. The current state of knowledge concerning the factors influencing T cells’ susceptibility to or resistance to immunotherapy was explored by Philip and Schietinger [29], who also highlighted unresolved areas of research.

Currently, there is a significant emphasis on comprehending diseases with high mortality rates on a global scale, including infectious diseases and cancer. To achieve this understanding, mathematical modeling proves to be a valuable tool for analyzing diseases that impact populations universally [30]. Cancer remains a significant health challenge, leading to the loss of human lives despite advancements in the scientific and medical fields. In recent times, the medical community has adopted protocols incorporating interleukin-10 (IL-10) and anti-PD-L1 inhibitors to enhance the immune system’s response against cancer cells [31]. Authors explore a recent biological model created to analyze the behavior of cancer cells, diabetes and smoking by using different fractional techniques given in [32–34] respectively.

The article [35] by Casiraghi and colleagues describes research that was carried out at a single medical facility with individuals who had received care during the preceding two decades. Their study reduced heterogeneity within the patient group by implementing a multimodality strategy and integrating the most recent staging methods. This approach facilitated the identification of potential prognostic factors for optimizing patient selection. Liang et al.’s study [36] focused on patients with Small Cell Lung Cancer (SCLC) who had undergone chemotherapy. Their primary objective was to develop a predictive nomogram. Chao and his research team conducted an investigation and developed a predictive algorithm [37] with the specific purpose of identifying individuals who could benefit from surgical intervention. Additionally, Li et al. [38] retrospectively examined 18 patients with Non-Small Cell Lung Cancer (NSCLC) who presented complex EGFR mutations, emphasizing mutations that encompass both common and uncommon genetic alterations. Different types of investigation on Cancer model are given in the references [39–41].

Several models have been developed to investigate the influence of different immune cell types on tumor cells. However, only a limited number of models have explored dendritic cells’ influence on tumor cells. Moreover, prior discussions have not considered surgical interventions as a viable treatment option. In contrast, our approach integrates both surgery and chemotherapy into our treatment regimen. Additionally, previous authors failed to establish a clear starting point for this treatment phase. In contrast, our research distinguishes itself by introducing a mathematical model for lung cancer that is not small-cell that incorporates the potential treatment options of chemotherapy and surgery, illustrating their combined impact. We also offer recommendations for the most effective treatment strategy among the various combinations of surgery and varying chemotherapy dosages. Finally, we propose a specific time frame for initiating treatment to improve a patient’s prognosis. Considering the significance of the aforementioned, we want to concentrate on these basic issues in this work, using a model that has been specially tailored to depict the dynamics that characterize lung cancer as well as the limitations of our response to it. Using a traditional TCD design that allows for lengthy incubation, we first presented the epidemic dynamics inside one community with a particular social pattern.

Here, the existing model to investigate cancer disease published in June 2023 is given in [15] as follows:

$$\begin{array}{ccc} \frac{d\;T}{d\;t} & = & \;\alpha \;T(1-\beta T)\;-\;\gamma \;T\;-\;\phi \;C\;T, \end{array}$$

$$\begin{array}{ccc} \frac{d\;C}{d\;t} & = & \;\nu \;T\;-\;\eta \;C\;T\;-\;\kappa C,\\ \frac{d\;D}{d\;t} & = & \;\mu \;+\;\sigma \;D\;T\;-\;\rho \;C\;D\;-\;\omega \;D. \end{array}$$ (1)

Initial conditions corresponds to the aforementioned system:

$$\begin{array}{c} T\left(\;0\right)\;=\;T^{0},\;\;C\left(0\right)\;=\;C^{0},\;\;D\left(0\right)\;=\;D^{0}. \end{array}$$

The main objective of this study is to incorporate innovative fractional derivatives into mathematical analysis and simulation for the improvement of the Lung Cancer model. Lung Cancer poses a considerable threat to human life due to its high level of danger. In the previous model given above has delimitations that lung cancer can not be controlled with dendritic cells. So, we introduce cytokine and the anti-PD-L1 inhibitor to boost up the low immune individuals. We developed new mathematical model by taking these two measures which helps to control lung cancer early which we shall observe on simulation easily. The research involves confirming the presence of a solution system with unique characteristics and conducting a qualitative evaluation of this system. Furthermore, the fractal fractional derivative is utilized to investigate the real-world behavior of the newly developed mathematical model. Finally, numerical simulations are used to reinforce and authenticate the biological findings.

**Definition 1.1**

If 0 < *ξ* ≤ 1 and 0 < λ ≤ 1, then the Riemann-Liouville operator for the Fractal-Fractional Operator (FFO) with Mittag-Leffler (ML) kernel is defined as *U*(*t*) [42].

$$\begin{array}{c} \begin{array}{c} FFM\\ 0 \end{array}D_{t}^{\xi ,\lambda }U\left(t\right)=\frac{AB(\xi )}{1-\xi }\int_{0}^{t}\frac{dU(\Omega )}{dt^{\lambda }}E_{\xi }[-\frac{\xi }{1-\xi }{\left(t-\Omega \right)}^{\xi }]d\Omega \;\;, \end{array}$$

involving 0 < *ξ*, λ ≤ 1 and $AB\left(\xi \right)=1-\xi +\frac{\xi }{\Gamma (\xi )}$.

Therefore, the function *U*(*t*), which has an order of (*ξ*, λ) and a ML kernel, is given as follows.

$$\begin{array}{c} \begin{array}{c} FFM\\ 0 \end{array}D_{t}^{\xi ,\lambda }U\left(t\right)=\frac{\lambda \left(1-\xi \right)t^{\lambda -1}U\left(t\right)}{AB(\xi )}+\frac{\xi \lambda }{AB(\xi )}\int_{0}^{t}\Omega^{\xi -1}\left(t-\Omega \right)U\left(\Omega \right)d\Omega \;\;. \end{array}$$

## 2 Formulation of TCD*IL*_2_Z model

A mathematical model is formulated for Lung Cancer by introducing *IL*_2_ cytokine and anti-PD-L1 inhibitor for treatment as well as for strong immune, whereas the previous model used the TCD framework which is not enough to control lung cancer. In this new model, the new model is referred to as TCD*IL*_2_Z, where “T” represents the tumor cells, “C” represents the CD8+ T cells, “D” represents the dendritic cells, “*IL*_2_” represents the cytokine and “Z” represents the anti-PD-L1 inhibitor.

**Fig 1:**
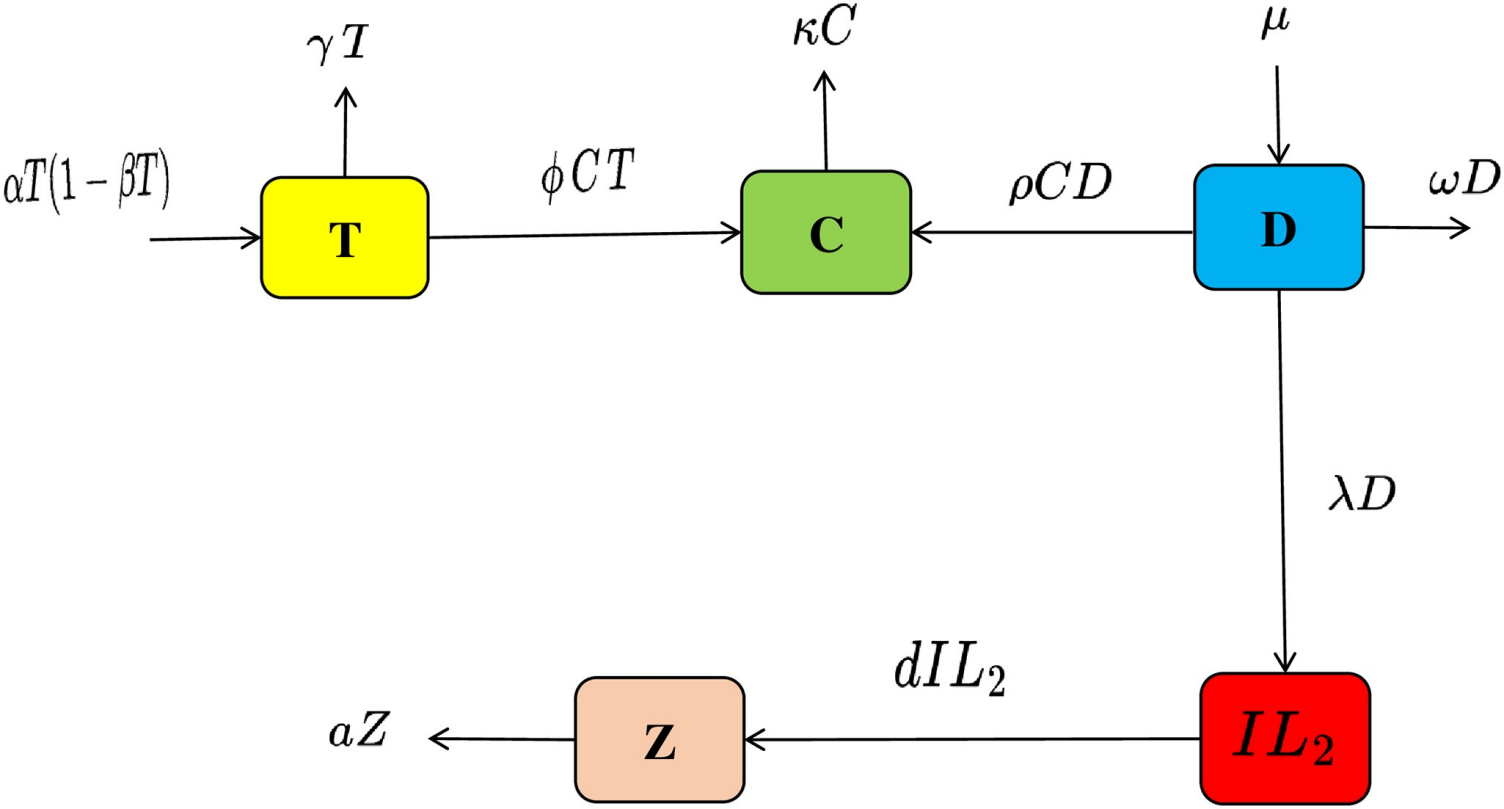
Flow chart. The flow chart illustrates for model formulation.

In this model, we introduce several key parameters: The expression “*αT*(1 − *βT*)” describes the logistic growth of the tumor, “*γ*” represents the constant rate at which tumor cells are destroyed by dendritic cells, “*ϕ*” signifies the rate at which CD8+ T cells eliminate tumor cells, “*κ*” denotes the natural death rate of CD8+ T cells, “*μ*” characterizes the sources responsible for generating dendritic cells, “*ρ*” symbolizes the rate at which dendritic cells are rendered inactive by CD8+ T cells, “*ω*” indicates the natural death rate of dendritic cells, “λ” represents the source of *IL*_2_ to reduce the dendritic cell’s, “*d*” represents the rate at which anti-PD-L1 inhibitor cell’s increases the immune system and “*a*” represents the natural death rate of anti-PD-L1 inhibitor. Fig 1 represents the flow chart for the newly developed model TCD*IL*_2_Z.

The model that was developed based on the generalized hypothesis with the *IL*_2_ cytokine and anti-PD-L1 inhibitor *Z* effect is presented as follows:

$$\begin{array}{ccc} \frac{d\;T}{d\;t} & = & \alpha T(1-\beta T)-\gamma T-\phi CT,\\ \frac{d\;C}{d\;t} & = & \phi CT+\rho CD-\kappa C,\\ \frac{d\;D}{d\;t} & = & \mu -\rho CD-\omega D-\lambda D,\\ \frac{d\;IL_{2}}{d\;t} & = & \lambda D-dIL_{2},\\ \frac{d\;Z}{d\;t} & = & dIL_{2}-aZ. \end{array}$$ (2)

The following are initial conditions linked with the described system:

$$\begin{array}{c} T\left(0\right)\;=\;T^{0},\;\;C\left(0\right)\;=\;C^{0},\;\;D\left(0\right)\;=\;D^{0},\;\;Il_{2}\left(0\right)\;=\;Il_{2}^{0},\;\;Z\left(0\right)\;=\;Z^{0}. \end{array}$$

Using FFO with Mittag-Lefller (ML) definition, above model becomes

$$\begin{array}{ccc} \begin{array}{c} FFM\\ 0 \end{array}D_{t}^{\xi ,\lambda }T\left(t\right) & = & \alpha T(1-\beta T)-\gamma T-\phi CT,\\ \begin{array}{c} FFM\\ 0 \end{array}D_{t}^{\xi ,\lambda }C\left(t\right) & = & \phi CT+\rho CD-\kappa C,\\ \begin{array}{c} FFM\\ 0 \end{array}D_{t}^{\xi ,\lambda }D\left(t\right) & = & \mu -\rho CD-\omega D-\lambda D,\\ \begin{array}{c} FFM\\ 0 \end{array}D_{t}^{\xi ,\lambda }IL_{2}\left(t\right) & = & \lambda D-dIL_{2},\\ \begin{array}{c} FFM\\ 0 \end{array}D_{t}^{\xi ,\lambda }Z\left(t\right) & = & dIL_{2}-aZ. \end{array}$$ (3)

Here $\begin{array}{c} FFM\\ 0 \end{array}D_{t}^{\xi ,\lambda }$, is the fractal fractional operator with Mittag-Lefller (FFM), where 0 < *ξ* ≤ 1 and 0 < λ ≤ 1. The following are initial conditions linked with the described system:

$$\begin{array}{c} T\left(0\right)\;=\;T^{0},\;\;C\left(0\right)\;=\;C^{0},\;\;D\left(0\right)\;=\;D^{0},\;\;Il_{2}\left(0\right)\;=\;Il_{2}^{0},\;\;Z\left(0\right)\;=\;Z^{0}. \end{array}$$

### 2.1 Equilibrium point and reproductive number

For this model, the point of equilibrium without disease is

$$\begin{array}{c} D_{1}\left(T,\;C,\;D,\;IL_{2},\;Z\right)=(0,0,\frac{\mu }{\lambda +\omega },\frac{\lambda \mu }{d(\lambda +\omega )},\frac{\lambda \mu }{a(\lambda +\omega )}). \end{array}$$

as well as the endemic point of equilibrium as follows $D_{2}\left(T^{*},C^{*},D^{*},IL_{2}^{*},Z^{*}\right)$. where

$$\begin{array}{ccc} T^{*} & = & \frac{\alpha \beta \kappa \rho +\alpha \rho \phi -\gamma \rho \phi +\lambda \phi^{2}+\phi^{2}\omega -\sqrt{4\alpha \beta \mu \rho^{2}\phi^{2}+{\left(\alpha \rho \left(-\beta \kappa +\phi \right)+\phi \left(-\gamma \rho +\phi \left(\lambda +\omega \right)\right)\right)}^{2}}}{2\alpha \beta \rho \phi }, \end{array}$$

$$\begin{array}{ccc} C^{*} & = & -\frac{\alpha \beta \kappa \rho -\alpha \rho \phi +\gamma \rho \phi +\lambda \phi^{2}+\phi^{2}\omega -\sqrt{4\alpha \beta \mu \rho^{2}\phi^{2}+{\left(\alpha \rho \left(-\beta \kappa +\phi \right)+\phi \left(-\gamma \rho +\phi \left(\lambda +\omega \right)\right)\right)}^{2}}}{2\rho \phi^{2}}, \end{array}$$

$$\begin{array}{ccc} D^{*} & = & \frac{\alpha \beta \kappa \rho -\alpha \rho \phi +\gamma \rho \phi -\lambda \phi^{2}-\phi^{2}\omega +\sqrt{4\alpha \beta \mu \rho^{2}\phi^{2}+{\left(\alpha \rho \left(-\beta \kappa +\phi \right)+\phi \left(-\gamma \rho +\phi \left(\lambda +\omega \right)\right)\right)}^{2}}}{2\alpha \beta \rho^{2}}, \end{array}$$

$$\begin{array}{ccc} IL_{2}^{*} & = & \frac{\lambda (\alpha \beta \kappa \rho -\alpha \rho \phi +\gamma \rho \phi -\lambda \phi^{2}-\phi^{2}\omega +\sqrt{4\alpha \beta \mu \rho^{2}\phi^{2}+{\left(\alpha \rho \left(-\beta \kappa +\phi \right)+\phi \left(-\gamma \rho +\phi \left(\lambda +\omega \right)\right)\right)}^{2}})}{2d\alpha \beta \rho^{2}}, \end{array}$$

$$\begin{array}{ccc} Z^{*} & = & \frac{\lambda (\alpha \beta \kappa \rho -\alpha \rho \phi +\gamma \rho \phi -\lambda \phi^{2}-\phi^{2}\omega +\sqrt{4\alpha \beta \mu \rho^{2}\phi^{2}+{\left(\alpha \rho \left(-\beta \kappa +\phi \right)+\phi \left(-\gamma \rho +\phi \left(\lambda +\omega \right)\right)\right)}^{2}})}{2a\alpha \beta \rho^{2}}. \end{array}$$

Reproductive number for the developed system is derived from next generation technique which represents that at how much rate the cancer spread in the community depending on the value of *R*_0_. After substituting the value of parameters we get that the system is disease free due to introducing new control variables.

$$\begin{array}{c} R_{0}=\frac{\mu \rho }{\kappa (\lambda +\omega )}<1. \end{array}$$

## 3 Bounded and positive solutions

In this section, we demonstrate the boundedness and positivity of the developed model.

**Theorem 3.1** The considered initial condition is as follows

$$\begin{array}{c} \{T^{0},C^{0},D^{0},IL_{2}^{0},Z^{0}\}\subset ϒ, \end{array}$$

therefore the solutions {*T*, *C*, *D*, *IL*_2_, *Z*} will be positive, ∀ t ≥ 0.

**Proof:** We will begin the primary analysis to show the improved quality of the solutions. These solutions effectively address real-world issues and have positive outcomes. We will follow the methodology provided in references [43–45]. In this segment, we will examine the conditions required to ensure positive outcomes from the newly developed model. To accomplish this, we will establish the standard.

$$\begin{array}{c} \|\zeta {\|}_{\infty }=\underset{t\in D_{\zeta }}{\text{sup}}\;|\zeta \left(t\right)|, \end{array}$$

here “*D*_*ζ*_” represents the *ζ* domain.

So, continue with *T*(*t*).

$$\begin{array}{ccc} \begin{array}{c} FFM\\ 0 \end{array}D_{t}^{\xi ,\lambda }T\left(t\right) & = & \alpha T(1-\beta T)-\gamma T-\phi CT,\;\forall t\geq 0,\\ & \geq & -(\alpha \beta |T|+\gamma +\phi |C|)T,\;\forall t\geq 0.\\ & \geq & -(\alpha \beta \;\underset{t\in D_{T}}{\text{sup}}\;|T|+\gamma +\phi \;\underset{t\in D_{C}}{\text{sup}}\;|C|)T,\;\forall t\geq 0.\\ & \geq & -(\alpha \beta \|T{\|}_{\infty }+\gamma +\phi \|C{\|}_{\infty })T,\;\forall t\geq 0. \end{array}$$

This outcome was achieved.

$$\begin{array}{c} T\left(t\right)\geq T\left(0\right)E_{\xi }[-\frac{b^{1-\lambda }\xi \left(\alpha \beta \|T{\|}_{\infty }+\gamma +\phi \|C{\|}_{\infty }\right)t^{\xi }}{AB\left(\xi \right)-\left(1-\xi \right)\left(\alpha \beta \|T{\|}_{\infty }+\gamma +\phi \|C{\|}_{\infty }\right)}],\;\forall t\geq 0, \end{array}$$

here “b” represents the time element. This demonstrates that the *T*(*t*) individuals must be positive ∀*t*≥ 0. Now, we have *C*(*t*) individuals as follows.

$$\begin{array}{ccc} \begin{array}{c} FFM\\ 0 \end{array}D_{t}^{\xi ,\lambda }C\left(t\right) & = & \phi CT+\rho CD-\kappa C,\;\forall \;\;t\geq 0,\\ & \geq & -(\kappa -\phi |T|-\rho |D|)C,\;\forall \;\;t\geq 0,\\ & \geq & -(\kappa -\phi \;\underset{t\in D_{T}}{\text{sup}}\;|T|-\rho \;\underset{t\in D_{D}}{\text{sup}}\;|D|)C,\;\forall \;\;t\geq 0,\\ & \geq & -(\kappa -\phi \|T{\|}_{\infty }-\rho \|D{\|}_{\infty })C,\;\forall \;\;t\geq 0. \end{array}$$

This outcome was achieved.

$$\begin{array}{c} C\left(t\right)\geq C\left(0\right)E_{\xi }[-\frac{b^{1-\lambda }\xi \left(\kappa -\phi \|T{\|}_{\infty }-\rho \|D{\|}_{\infty }\right)t^{\xi }}{AB\left(\xi \right)-\left(1-\xi \right)\left(\kappa -\phi \|T{\|}_{\infty }-\rho \|D{\|}_{\infty }\right)}],\;\forall \;\;t\geq 0, \end{array}$$

here “b” represents the time element. This demonstrates that the *C*(*t*) individuals must be positive ∀ *t*≥ 0. Now, we have *D*(*t*) individuals as follows.

$$\begin{array}{ccc} \begin{array}{c} FFM\\ 0 \end{array}D_{t}^{\xi ,\lambda }D\left(t\right) & = & \mu -\rho CD-\omega D-\lambda D,\;\forall t\geq 0,\\ & \geq & -(\rho |C|+\omega +\lambda )D,\;\forall t\geq 0,\\ & \geq & -(\rho \;\underset{t\in D_{C}}{\text{sup}}\;|C|+\omega +\lambda )D,\;\forall t\geq 0,\\ & \geq & -(\rho \|C{\|}_{\infty }+\omega +\lambda )D,\;\forall t\geq 0. \end{array}$$

This outcome was achieved.

$$\begin{array}{c} D\left(t\right)\geq D\left(0\right)E_{\xi }[-\frac{b^{1-\lambda \xi }\left(\rho \|C{\|}_{\infty }+\omega +\lambda \right)t^{\xi }}{AB\left(\xi \right)-\left(1-\xi \right)(\rho \|C{\|}_{\infty }+\omega +\lambda )}],\;\forall t\geq 0, \end{array}$$

here “b” represents the time element. This demonstrates that the *D*(*t*) individuals must be positive ∀ *t*≥ 0. Now, we have *IL*_2_(*t*) individuals as follows.

$$\begin{array}{ccc} \begin{array}{c} FFM\\ 0 \end{array}D_{t}^{\xi ,\lambda }IL_{2}\left(t\right) & = & \lambda D-dIL_{2},\;\forall t\geq 0,\\ & \geq & -dIL_{2},\;\forall t\geq 0. \end{array}$$

This outcome was achieved.

$$\begin{array}{c} IL_{2}\left(t\right)\geq IL_{2}\left(0\right)E_{\xi }[-\frac{b^{1-\lambda }\xi \left(d\right)t^{\xi }}{AB(\xi )-(1-\xi )(d)}],\;\forall t\geq 0, \end{array}$$

here “b” represents the time element. This demonstrates that the *IL*_2_(*t*) individuals must be positive ∀ *t*≥ 0. Now, we have *Z*(*t*) individuals as follows.

$$\begin{array}{ccc} \begin{array}{c} FFM\\ 0 \end{array}D_{t}^{\xi ,\lambda }Z\left(t\right) & = & dIL_{2}-aZ,\;\forall \;t\;\geq \;0,\\ & \geq & -aZ(t),\;\forall \;t\;\geq \;0. \end{array}$$

This outcome was achieved.

$$\begin{array}{c} Z\left(t\right)\geq Z\left(0\right)E_{\xi }[-\frac{b^{1-\lambda }\xi \left(a\right)t^{\xi }}{AB(\xi )-(1-\xi )(a)}],\;\forall t\geq 0, \end{array}$$

here “b” represents the time element. This demonstrates that the *Z*(*t*) individuals must be positive ∀ *t*≥ 0.

**Theorem 3.2** Solutions of our developed model given in Eq (3) with positive initial values are all bounded.

**Proof:** Above theorem demonstrates that the solutions of our developed model must be positive ∀ *t* ≥ 0, and strategies are described in [46]. Because *X* = *T* + *C* + *D*. Therefore, given as follows.

$$\begin{array}{c} \begin{array}{c} FFM\\ 0 \end{array}D_{t}^{\xi ,\lambda }X\left(t\right)=a-\delta X-\left(\mu_{0}+\omega -b+\phi \right)I. \end{array}$$

We achieved this

$$\begin{array}{c} \Psi_{p}=\left\{T,\;C,\;D\in R_{+}^{3}|T+D\leq X\right\}\;\forall \;\;t\geq 0. \end{array}$$

It has also as *X*_*υ*_ = *IL*_2_ + *Z*. Therefore, we have developed.

$$\begin{array}{c} \begin{array}{c} FFM\\ 0 \end{array}D_{t}^{\xi ,\lambda }X_{\upsilon }\left(t\right)=\lambda D-aZ+X_{\upsilon }-X_{\upsilon }, \end{array}$$

on solving above equation and taking *t* → ∞, we get

$$\begin{array}{c} X_{\upsilon }\leq \lambda D-aZ+X_{\upsilon }. \end{array}$$

Thus

$$\begin{array}{c} \Psi_{\upsilon }=\{IL_{2},\;\;Z\in R_{+}^{2}|X_{\upsilon }\leq \lambda D-aZ+X_{\upsilon }\}\;\forall \;\;t\geq 0. \end{array}$$

The model’s mathematical solutions (3) are confined to region Ψ.

$$\begin{array}{c} \Psi =\{T,\;\;C,\;\;D,\;\;IL_{2},\;\;Z\in R_{+}^{5}|T+D\leq X,X_{\upsilon }\leq \lambda D-aZ+X_{\upsilon }\}\;\forall \;\;t\geq 0. \end{array}$$

This demonstrates that for every *t* ≥ 0, all solutions remain positive consistent with provided initial circumstances in domain Ψ.

**Theorem 3.3** The newly developed Lung Cancer model Eq (3) in $R_{+}^{5}$ is unique and constrained, in addition to the initial circumstance.

**Proof:** In this particular scenario, we applied the procedure described in [46]. We’ve got

$$\begin{array}{ccc} \begin{array}{c} FFM\\ 0 \end{array}D_{t}^{\xi ,\lambda }{\left(T\left(t\right)\right)}_{T=0} & = & \alpha T\;\geq 0,\\ \begin{array}{c} FFM\\ 0 \end{array}D_{t}^{\xi ,\lambda }{\left(C\left(t\right)\right)}_{C=0} & = & \phi CT+\rho CD\;\geq 0,\\ \begin{array}{c} FFM\\ 0 \end{array}D_{t}^{\xi ,\lambda }{\left(D\left(t\right)\right)}_{D=0} & = & \mu \;\geq 0,\\ \begin{array}{c} FFM\\ 0 \end{array}D_{t}^{\xi ,\lambda }{\left(IL_{2}\left(t\right)\right)}_{IL_{2}=0} & = & \lambda D\;\geq 0,\\ \begin{array}{c} FFM\\ 0 \end{array}D_{t}^{\xi ,\lambda }{\left(Z\left(t\right)\right)}_{Z=0} & = & dIL_{2}\;\geq 0. \end{array}$$ (4)

If $\left(T^{0},C^{0},D^{0},IL_{2}^{0},Z^{0}\right)$ ∈ $R_{+}^{5}$, then our obtain solution is unable to escape from the hyperplane, as stated in Eq (4). This proves that $R_{+}^{5}$ domain become a positive invariant.

## 4 Impact of global derivatives for uniqueness and exitance of solution

Riemann-Stieltjes integral has been widely recognized in the literature as the most commonly used integral. If

$$\begin{array}{c} Y(x)=\int y(x)dx. \end{array}$$

The Riemann-Stieltjes integral is given as follows.

$$\begin{array}{c} Y_{w}\left(x\right)=\int y\left(x\right)dw\left(x\right), \end{array}$$

*y*(*x*) global derivative with regard to *w*(*x*) is

$$\begin{array}{c} D_{w}y\left(x\right)=\underset{h\to 0}{\text{lim}}\;\frac{y(x+h)-y(x)}{w(x+h)-w(x)}. \end{array}$$

If the above functions numerator and denominator differentiated, then we get

$$\begin{array}{c} D_{w}y\left(x\right)=\frac{y^{\prime }\left(x\right)}{w^{\prime }\left(x\right)}, \end{array}$$

assuming that *w*′(*x*) ≠ 0, ∀*x* ∈ *D*_*w*′_. Now, we will test the impact on Corona virus by using the global derivative instead of classical derivative.

$$\begin{array}{ccc} D_{w}T & = & \alpha T(1-\beta T)-\gamma T-\phi CT,\\ D_{w}C & = & \phi CT+\rho CD-\kappa C,\\ D_{w}D & = & \mu -\rho CD-\omega D-\lambda D,\\ D_{w}IL_{2} & = & \lambda D-dIL_{2},\\ D_{w}Z & = & dIL_{2}-aZ. \end{array}$$

For the purpose of cleanliness, we shall suppose that w is differentiable.

$$\begin{array}{ccc} T^{\prime } & = & w^{\prime }\left[\alpha T\left(1-\beta T\right)-\gamma T-\phi CT\right],\\ C^{\prime } & = & w^{\prime }\left[\phi CT+\rho CD-\kappa C\right],\\ D^{\prime } & = & w^{\prime }\left[\mu -\rho CD-\omega D-\lambda D\right],\\ IL_{2}^{\prime } & = & w^{\prime }\left[\lambda D-dIL_{2}\right],\\ Z^{\prime } & = & w^{\prime }\left[dIL_{2}-aZ\right]. \end{array}$$

An appropriate choice of the function *w*(*t*) will lead to a specific outcome. For instance, if *w*(*t*) = *t*^*α*^, where *α* is a real number, we will observe fractal movement. We had to take action due to the circumstances that

$$\begin{array}{c} \|w^{\prime }{\|}_{\infty }=\;\underset{t\in D_{w^{\prime }}}{\text{sup}}\;|w^{\prime }\left(t\right)|<N. \end{array}$$

The below example demonstrate the unique solution for the developed system.

$$\begin{array}{ccc} T^{\prime } & = & w^{\prime }\left[\alpha T\left(1-\beta T\right)-\gamma T-\phi CT\right]=X_{1}\left(t,T,G\right),\\ C^{\prime } & = & w^{\prime }\left[\phi CT+\rho CD-\kappa C\right]=X_{2}\left(t,T,G\right),\\ D^{\prime } & = & w^{\prime }\left[\mu -\rho CD-\omega D-\lambda D\right]=X_{3}\left(t,T,G\right),\\ IL_{2}^{\prime } & = & w^{\prime }\left[\lambda D-dIL_{2}\right]=X_{4}\left(t,T,G\right),\\ Z^{\prime } & = & w^{\prime }\left[dIL_{2}-aZ\right]=X_{5}\left(t,T,G\right). \end{array}$$

where *G* = *C*, *D*, *IL*_2_, *Z*.

We need to confirm the first two requirements as follows.

**1.** ∣*X*(*t*, *T*, *G*)∣^2^ < *K*(1 + ∣*T*∣^2^,
**2.** ∀ *T*_1_, *T*_2_, we have, $\|X\left(t,T_{1},G\right)-X\left(t,T_{2},G\right){\|}^{2}<\bar{K}\|T_{1}-T_{2}{\|}_{\infty }^{2}$.

Initially,

$$\begin{array}{ccc} |X_{1}\left(t,T,G\right){|}^{2} & = & |w^{\prime }[\alpha T(1-\beta T)-\gamma T-\phi CT]{|}^{2},\\ & = & |w^{\prime }[\alpha T+(-\alpha \beta T-\gamma -\phi C)T]{|}^{2},\\ & \leq & 2|w^{\prime }{|}^{2}(|\alpha T{|}^{2}+|(-\alpha \beta T-\gamma -\phi C)T{|}^{2}),\\ & \leq & 2\;\underset{t\in D_{w^{\prime }}}{\text{sup}}\;|w^{\prime }{|}^{2}\alpha^{2}\;\underset{t\in D_{T}}{\text{sup}}\;|T{|}^{2}+6\;\underset{t\in D_{w^{\prime }}}{\text{sup}}\;|w^{\prime }{|}^{2}(\alpha^{2}\beta^{2}\;\underset{t\in D_{T}}{\text{sup}}\;|T{|}^{2}+\gamma^{2}+\phi^{2}\;\underset{t\in D_{C}}{\text{sup}}\;|C{|}^{2})\\ & \times & |T{|}^{2},\\ & \leq & 2\|w^{\prime }{\|}_{\infty }^{2}\alpha^{2}\|T{\|}_{\infty }^{2}+6\|w^{\prime }{\|}_{\infty }^{2}(\alpha^{2}\beta^{2}\|T{\|}_{\infty }^{2}+\gamma^{2}+\phi^{2}\|C{\|}_{\infty }^{2})|T{|}^{2},\\ & \leq & 2\|w^{\prime }{\|}_{\infty }^{2}\alpha^{2}\|T{\|}_{\infty }^{2}(1+\frac{3}{\alpha^{2}\|T{\|}_{\infty }^{2}}(\alpha^{2}\beta^{2}\|T{\|}_{\infty }^{2}+\gamma^{2}+\phi^{2}\|C{\|}_{\infty }^{2})|T{|}^{2}),\\ & \leq & K_{1}\left(\;1\;+\;|\;T\;{|}^{2}\right). \end{array}$$

under the condition

$$\begin{array}{c} \frac{3}{\;\alpha^{2}\|\;T{\|}_{\;\infty }^{2}}(\;\alpha^{2}\;\beta^{2}\|\;T{\|}_{\;\infty }^{2}\;+\;\gamma^{2}\;+\;\phi^{2}\|C{\|}_{\;\infty }^{2})\;<\;1, \end{array}$$

involving

$$\begin{array}{c} K_{1}=2\|w^{\prime }{\|}_{\infty }^{2}\alpha^{2}\|T{\|}_{\infty }^{2}. \end{array}$$

$$\begin{array}{ccc} |X_{2}\left(t,T,G\right){|}^{2} & = & |w^{\prime }[\phi CT+\rho CD-\kappa C]{|}^{2},\\ & = & |w^{\prime }[(-\kappa C)+(\phi T+\rho D)C]{|}^{2},\\ & \leq & 2|w^{\prime }{|}^{2}(|\left(-\kappa C\right){|}^{2}+|\left(\phi T+\rho D\right)C{|}^{2}),\\ & \leq & 2\;\underset{t\in D_{w^{\prime }}}{\text{sup}}\;|w^{\prime }{|}^{2}\kappa^{2}\;\underset{t\in D_{C}}{\text{sup}}\;|C{|}^{2}+4\;\underset{t\in D_{w^{\prime }}}{\text{sup}}\;|w^{\prime }{|}^{2}\left(\phi^{2}\;\underset{t\in D_{T}}{\text{sup}}\;|T{|}^{2}+\rho^{2}\;\underset{t\in D_{D}}{\text{sup}}\;|D{|}^{2}\right)|C{|}^{2},\\ & \leq & 2\|w^{\prime }{\|}_{\infty }^{2}\kappa^{2}\|C{\|}_{\infty }^{2}+4\|w^{\prime }{\|}_{\infty }^{2}\left(\phi^{2}\|T{\|}_{\infty }^{2}+\rho^{2}\|D{\|}_{\infty }^{2}\right)|C{|}^{2},\\ & \leq & 2\|w^{\prime }{\|}_{\infty }^{2}\kappa^{2}\|C{\|}_{\infty }^{2}(1+\frac{2\left(\phi^{2}\|T{\|}_{\infty }^{2}+\rho^{2}\|D{\|}_{\infty }^{2}\right)|C{|}^{2}}{\kappa^{2}\|C{\|}_{\infty }^{2}}),\\ & \leq & K_{2}\left(1+|C{|}^{2}\right). \end{array}$$

under the condition

$$\begin{array}{c} \frac{2(\phi^{2}\|T{\|}_{\infty }^{2}+\rho^{2}\|D{\|}_{\infty }^{2})}{\kappa^{2}\|C{\|}_{\infty }^{2}}<1, \end{array}$$

where

$$\begin{array}{c} K_{2}=2\|w^{\prime }{\|}_{\infty }^{2}\kappa^{2}\|C{\|}_{\infty }^{2}. \end{array}$$

$$\begin{array}{ccc} |X_{3}\left(t,T,G\right){|}^{2} & = & |w^{\prime }[\mu -\rho CD-\omega D-\lambda D]{|}^{2},\\ & = & |w^{\prime }[\mu +(-\rho C-\omega -\lambda )D]{|}^{2},\\ & \leq & 2|w^{\prime }{|}^{2}(|\mu {|}^{2}+|\left(-\rho C-\omega -\lambda \right)D{|}^{2}),\\ & \leq & 2\;\underset{t\in D_{w^{\prime }}}{\text{sup}}\;|w^{\prime }{|}^{2}\mu^{2}+6\;\underset{t\in D_{w^{\prime }}}{\text{sup}}\;|w^{\prime }{|}^{2}[\rho^{2}\;\underset{t\in D_{C}}{\text{sup}}\;|C{|}^{2}+\omega^{2}+\lambda^{2}]|D{|}^{2},\\ & \leq & 2\||w^{\prime }{\|}_{\infty }^{2}\mu^{2}+6\||w^{\prime }{\|}_{\infty }^{2}[\rho^{2}\|C{\|}_{\infty }^{2}+\omega^{2}+\lambda^{2}]|D{|}^{2},\\ & \leq & 2\|w^{\prime }{\|}_{\infty }^{2}\mu^{2}(1+\frac{3}{\mu^{2}}(\rho^{2}\|C{\|}_{\infty }^{2}+\omega^{2}+\lambda^{2})|D{|}^{2}),\\ & \leq & K_{3}\left(1+|D{|}^{2}\right). \end{array}$$

under the condition

$$\begin{array}{c} \frac{3}{\mu^{2}}(\rho^{2}\|C{\|}_{\infty }^{2}+\omega^{2}+\lambda^{2})<1, \end{array}$$

where

$$\begin{array}{c} K_{3}=2\||w^{\prime }{\|}_{\infty }^{2}\mu^{2}. \end{array}$$

$$\begin{array}{ccc} |X_{4}\left(t,T,G\right){|}^{2} & = & |w^{\prime }[\lambda D-dIL_{2}]{|}^{2},\\ & = & |w^{\prime }[\lambda D+(-dIL_{2})]{|}^{2},\\ & \leq & 2|w^{\prime }{|}^{2}(|\lambda D{|}^{2}+|\left(-dIL_{2}\right){|}^{2}),\\ & \leq & 2\;\underset{t\in D_{w^{\prime }}}{\text{sup}}\;|w^{\prime }{|}^{2}\lambda^{2}\;\underset{t\in D_{D}}{\text{sup}}\;|D{|}^{2}+2\;\underset{t\in D_{w^{\prime }}}{\text{sup}}\;|w^{\prime }{|}^{2}d^{2}|IL_{2}{|}^{2},\\ & \leq & 2\|w^{\prime }{\|}_{\infty }^{2}\lambda^{2}\|D{\|}_{\infty }^{2}+2\|w^{\prime }{\|}_{\infty }^{2}d^{2}|IL_{2}{|}^{2},\\ & \leq & 2\|w^{\prime }{\|}_{\infty }^{2}\lambda^{2}\|D{\|}_{\infty }^{2}(1+\frac{d^{2}|IL_{2}{|}^{2}}{\lambda^{2}\|D{\|}_{\infty }^{2}}),\\ & \leq & K_{4}\left(1+|IL_{2}{|}^{2}\right). \end{array}$$

under the condition

$$\begin{array}{c} \frac{d^{2}}{\lambda^{2}\|D{\|}_{\infty }^{2}}<1, \end{array}$$

where

$$\begin{array}{c} K_{4}=2\|w^{\prime }{\|}_{\infty }^{2}\lambda^{2}\|D{\|}_{\infty }^{2}. \end{array}$$

$$\begin{array}{ccc} |X_{5}\left(t,T,G\right){|}^{2} & = & |w^{\prime }[dIL_{2}-aZ]{|}^{2},\\ & = & |w^{\prime }[dIL_{2}+\left(-aZ\right)]{|}^{2},\\ & \leq & 2|w^{\prime }{|}^{2}(|dIL_{2}{|}^{2}+|\left(-aZ\right){|}^{2}),\\ & \leq & 2\;\underset{t\in D_{w^{\prime }}}{\text{sup}}\;|w^{\prime }{|}^{2}(d^{2}\;\underset{t\in D_{IL_{2}}}{\text{sup}}\;|IL_{2}{|}^{2})+2\;\underset{t\in D_{w^{\prime }}}{\text{sup}}\;|w^{\prime }{|}^{2}a^{2}|Z{|}^{2},\\ & \leq & 2\|w^{\prime }{\|}_{\infty }^{2}(d^{2}\|IL_{2}{\|}_{\infty }^{2})+2\|w^{\prime }{\|}_{\infty }^{2}a^{2}|Z{|}^{2},\\ & \leq & 2\|w^{\prime }{\|}_{\infty }^{2}(d^{2}\|IL_{2}{\|}_{\infty }^{2})(1+\frac{a^{2}|Z{|}^{2}}{d^{2}\|IL_{2}{\|}_{\infty }^{2}}),\\ & \leq & K_{5}\left(1+|Z{|}^{2}\right). \end{array}$$

under the circumstances

$$\begin{array}{c} \frac{a^{2}}{d^{2}\|IL_{2}{\|}_{\infty }^{2}}<1, \end{array}$$

where

$$\begin{array}{c} K_{5}=4\|w^{\prime }{\|}_{\infty }^{2}(d^{2}\|IL_{2}{\|}_{\infty }^{2}). \end{array}$$

Hence, linear growth criteria is satisfied.

Further, we verify Lipschitz condition as follows.

If

$$\begin{array}{c} |X_{1}\left(t,\;\;T_{1},\;\;C,\;\;D,\;\;IL_{2},\;\;Z\right)-X_{1}\left(t,\;\;T_{2},\;\;C,\;\;D,\;\;IL_{2},\;\;Z\right){|}^{2}\\ =|w^{\prime }[\left(\alpha +\alpha \beta T_{2}\right)+\left(-\alpha \beta T_{1}-\gamma -\phi C\right)]\left(T_{1}-T_{2}\right){|}^{2}, \end{array}$$

$$\begin{array}{c} |X_{1}\left(t,\;\;T_{1},\;\;C,\;\;D,\;\;IL_{2},\;\;Z\right)-X_{1}\left(t,\;\;T_{2},\;\;C,\;\;D,\;\;IL_{2},\;\;Z\right){|}^{2}\leq 2\\ |w^{\prime }{|}^{2}(2\left(\alpha^{2}+\alpha^{2}\beta^{2}|T_{2}{|}^{2}\right)+3(\alpha^{2}\beta^{2}|T_{1}{|}^{2}+\gamma^{2}+\phi^{2}|C{|}^{2}))|T_{1}-T_{2}{|}^{2}, \end{array}$$

$$\begin{array}{c} \underset{t\in D_{T}}{\text{sup}}\;|X_{1}\left(t,\;\;T_{1},\;\;C,\;\;D,\;\;IL_{2},\;\;Z\right)-X_{1}\left(t,\;\;T_{2},\;\;C,\;\;D,\;\;IL_{2},\;\;Z\right){|}^{2}\leq 2\;\underset{t\in D_{w^{\prime }}}{\text{sup}}\;|w^{\prime }{|}^{2}\\ (2\left(\alpha^{2}+\alpha^{2}\beta^{2}\;\underset{t\in D_{T_{2}}}{\text{sup}}\;|T_{2}{|}^{2}\right)+3(\alpha^{2}\beta^{2}\;\underset{t\in D_{T_{1}}}{\text{sup}}\;|T_{1}{|}^{2}+\gamma^{2}+\phi^{2}\;\underset{t\in D_{C}}{\text{sup}}\;|C{|}^{2}))\;\underset{t\in D_{T}}{\text{sup}}\;|T_{1}-T_{2}{|}^{2}, \end{array}$$

$$\begin{array}{c} \|X_{1}\left(t,\;\;T_{1},\;\;C,\;\;D,\;\;IL_{2},\;\;Z\right)-X_{1}\left(t,\;\;T_{2},\;\;C,\;\;D,\;\;IL_{2},\;\;Z\right){\|}_{\infty }^{2}\leq 2\|w^{\prime }{\|}_{\infty }^{2}\\ (2\left(\alpha^{2}+\alpha^{2}\beta^{2}\|T_{2}{\|}_{\infty }^{2}\right)+3\left(\alpha^{2}\beta^{2}\|T_{1}{\|}_{\infty }^{2}+\gamma^{2}+\phi^{2}\|C{\|}_{\infty }^{2}\right))\|T_{1}-T_{2}{\|}_{\infty }^{2}, \end{array}$$

$$\begin{array}{ccc} \|X_{1}\left(t,\;\;T_{1},\;\;C,\;\;D,\;\;IL_{2},\;\;Z\right)-X_{1}\left(t,\;\;T_{2},\;\;C,\;\;D,\;\;IL_{2},\;\;Z\right){\|}_{\infty }^{2} & \leq & \bar{K_{1}}\|T_{1}-T_{2}{\|}_{\infty }^{2}, \end{array}$$

where

$$\begin{array}{c} \bar{K_{1}}=2\|w^{\prime }{\|}_{\infty }^{2}(2\left(\alpha^{2}+\alpha^{2}\beta^{2}\|T_{2}{\|}_{\infty }^{2}\right)+3\left(\alpha^{2}\beta^{2}\|T_{1}{\|}_{\infty }^{2}+\gamma^{2}+\phi^{2}\|C{\|}_{\infty }^{2}\right)). \end{array}$$

If

$$\begin{array}{c} |X_{2}\left(t,\;\;T,\;\;C_{1},\;\;D,\;\;IL_{2},\;\;Z\right)-X_{2}\left(t,\;\;T,\;\;C_{2},\;\;D,\;\;IL_{2},\;\;Z\right){|}^{2}=|w^{\prime }\left(\phi CT+\rho CD-\kappa C\right){|}^{2}, \end{array}$$

$$\begin{array}{c} |X_{2}\left(t,\;\;T,\;\;C_{1},\;\;D,\;\;IL_{2},\;\;Z\right)-X_{2}\left(t,\;\;T,\;\;C_{2},\;\;D,\;\;IL_{2},\;\;Z\right){|}^{2}\leq |w^{\prime }{|}^{2}\\ \left(3\phi^{2}|T{|}^{2}+3\rho^{2}|D{|}^{2}+3\kappa^{2}\right)|C_{1}-C_{2}{|}^{2}, \end{array}$$

$$\begin{array}{c} \underset{t\in D_{C}}{\text{sup}}\;|X_{2}\left(t,\;\;T,\;\;C_{1},\;\;D,\;\;IL_{2},\;\;Z\right)-X_{2}\left(t,\;\;T,\;\;C_{2},\;\;D,\;\;IL_{2},\;\;Z\right){|}^{2}\leq \\ \;\underset{t\in D_{w^{\prime }}}{\text{sup}}\;|w^{\prime }{|}^{2}(3\phi^{2}\;\underset{t\in D_{T}}{\text{sup}}\;|T{|}^{2}+3\rho^{2}\;\underset{t\in D_{D}}{\text{sup}}\;|D{|}^{2}+3\kappa^{2})\times \;\underset{t\in D_{C}}{\text{sup}}\;|C_{1}-C_{2}{|}^{2}, \end{array}$$

$$\begin{array}{c} \|X_{2}\left(t,\;\;T,\;\;C_{1},\;\;D,\;\;IL_{2},\;\;Z\right)-X_{2}\left(t,\;\;T,\;\;C_{2},\;\;D,\;\;IL_{2},\;\;Z\right){\|}_{\infty }^{2}\leq \|w^{\prime }{\|}_{\infty }^{2}\\ \left(3\phi^{2}\|T{\|}_{\infty }^{2}+3\rho^{2}\|D{\|}_{\infty }^{2}+3\kappa^{2}\right)\|C_{1}-C_{2}{\|}_{\infty }^{2}, \end{array}$$

$$\begin{array}{c} \|X_{2}\left(t,\;\;T,\;\;C_{1},\;\;D,\;\;IL_{2},\;\;Z\right)-X_{2}\left(t,\;\;T,\;\;C_{2},\;\;D,\;\;IL_{2},\;\;Z\right){\|}_{\infty }^{2}\leq \bar{K_{2}}\|C_{1}-C_{2}{\|}_{\infty }^{2}, \end{array}$$

where

$$\begin{array}{c} \bar{K_{2}}=\|w^{\prime }{\|}_{\infty }^{2}\left(3\phi^{2}\|T{\|}_{\infty }^{2}+3\rho^{2}\|D{\|}_{\infty }^{2}+3\kappa^{2}\right). \end{array}$$

If

$$\begin{array}{c} |X_{3}\left(t,\;\;T,\;\;C,\;\;D_{1},\;\;IL_{2},\;\;Z\right)-X_{3}\left(t,\;\;T,\;\;C,\;\;D_{2},\;\;IL_{2},\;\;Z\right){|}^{2}=|w^{\prime }(-\rho C-\omega -\lambda ))\left(D_{1}-D_{2}\right){|}^{2}, \end{array}$$

$$\begin{array}{c} |X_{3}\left(t,\;\;T,\;\;C,\;\;D_{1},\;\;IL_{2},\;\;Z\right)-X_{3}\left(t,\;\;T,\;\;C,\;\;D_{2},\;\;IL_{2},\;\;Z\right){|}^{2}\leq \\ |w^{\prime }{|}^{2}(3\rho^{2}|C{|}^{2}+3\omega^{2}+3\lambda^{2})|D_{1}-D_{2}{|}^{2}, \end{array}$$

$$\begin{array}{c} \underset{t\in D_{D}}{\text{sup}}\;|X_{3}\left(t,\;\;T,\;\;C,\;\;D_{1},\;\;IL_{2},\;\;Z\right)-X_{3}\left(t,\;\;T,\;\;C,\;\;D_{2},\;\;IL_{2},\;\;Z\right){|}^{2}\leq \\ \underset{t\in D_{w^{\prime }}}{\text{sup}}\;|w^{\prime }{|}^{2}(3\rho^{2}|C{|}^{2}\;\underset{t\in D_{C}}{\text{sup}}\;|C{|}^{2}+3\omega^{2}+3\lambda^{2})\;\underset{t\in D_{D}}{\text{sup}}\;|D_{1}-D_{2}{|}^{2}, \end{array}$$

$$\begin{array}{c} \|X_{3}\left(t,\;\;T,\;\;C,\;\;D_{1},\;\;IL_{2},\;\;Z\right)-X_{3}\left(t,\;\;T,\;\;C,\;\;D_{2},\;\;IL_{2},\;\;Z\right){\|}_{\infty }^{2}\leq \\ \|w^{\prime }{\|}_{\infty }^{2}(3\rho^{2}\|C{\|}_{\infty }^{2}+3\omega^{2}+3\lambda^{2}))\|D_{1}-D_{2}{\|}_{\infty }^{2}, \end{array}$$

$$\begin{array}{c} \|X_{3}\left(t,\;\;T,\;\;C,\;\;D_{1},\;\;IL_{2},\;\;Z\right)-X_{3}\left(t,\;\;T,\;\;C,\;\;D_{2},\;\;IL_{2},\;\;Z\right){\|}_{\infty }^{2}\leq \bar{K_{3}}\|D_{1}-D_{2}{\|}_{\infty }^{2}, \end{array}$$

where

$$\begin{array}{c} \bar{K_{3}}=\|w^{\prime }{\|}_{\infty }^{2}(3\rho^{2}\|C{\|}_{\infty }^{2}+3\omega^{2}+3\lambda^{2})). \end{array}$$

If

$$\begin{array}{c} |X_{4}\left(t,\;\;T,\;\;C,\;\;D,\;\;{IL_{2}}_{1},\;\;Z\right)-X_{4}\left(t,\;\;T,\;\;C,\;\;D,\;\;{IL_{2}}_{2},\;\;Z\right){|}^{2}=|w^{\prime }\left(-d\right)\left({IL_{2}}_{1}-{IL_{2}}_{2}\right){|}^{2}, \end{array}$$

$$\begin{array}{c} |X_{4}\left(t,\;\;T,\;\;C,\;\;D,\;\;{IL_{2}}_{1},\;\;Z\right)-X_{4}\left(t,\;\;T,\;\;C,\;\;D,\;\;{IL_{2}}_{2},\;\;Z\right){|}^{2}=|w^{\prime }{|}^{2}\left(d^{2}\right)|{IL_{2}}_{1}-{IL_{2}}_{2}{|}^{2}, \end{array}$$

$$\begin{array}{c} \underset{t\in D_{IL_{2}}}{\text{sup}}X_{4}\left(t,\;\;T,\;\;C,\;\;D,\;\;{IL_{2}}_{1},\;\;Z\right)-X_{4}\left(t,\;\;T,\;\;C,\;\;D,\;\;{IL_{2}}_{2},\;\;Z\right){|}^{2}=\\ \underset{t\in D_{w^{\prime }}}{\text{sup}}\;|w^{\prime }{|}^{2}\left(d^{2}\right)\;\underset{t\in D_{IL_{2}}}{\text{sup}}\;|{IL_{2}}_{1}-{IL_{2}}_{2}{|}^{2}, \end{array}$$

$$\begin{array}{c} \|X_{4}\left(t,\;\;T,\;\;C,\;\;D,\;\;{IL_{2}}_{1},\;\;Z\right)-X_{4}\left(t,\;\;T,\;\;C,\;\;D,\;\;{IL_{2}}_{2},\;\;Z\right){\|}_{\infty }^{2}\leq \|w^{\prime }{\|}_{\infty }^{2}\left(d^{2}\right)\|{IL_{2}}_{1}-{IL_{2}}_{2}{\|}_{\infty }^{2}, \end{array}$$

$$\begin{array}{c} \|X_{4}\left(t,\;\;T,\;\;C,\;\;D,\;\;{IL_{2}}_{1},\;\;Z\right)-X_{4}\left(t,\;\;T,\;\;C,\;\;D,\;\;{IL_{2}}_{2},\;\;Z\right){\|}_{\infty }^{2}\leq \bar{K_{4}}\|{IL_{2}}_{1}-{IL_{2}}_{2}{\|}_{\infty }^{2}, \end{array}$$

where

$$\begin{array}{c} \bar{K_{4}}=\|w^{\prime }{\|}_{\infty }^{2}\left(d^{2}\right). \end{array}$$

If

$$\begin{array}{c} |X_{5}\left(t,\;\;T,\;\;C,\;\;D,\;\;IL_{2},\;\;Z_{1}\right)-X_{5}\left(t,\;\;T,\;\;C,\;\;D,\;\;IL_{2},\;\;Z_{2}\right){|}^{2}=|w^{\prime }\left(-a\right)\left(Z_{1}-Z_{2}\right){|}^{2}, \end{array}$$

$$\begin{array}{c} |X_{5}\left(t,\;\;T,\;\;C,\;\;D,\;\;IL_{2},\;\;Z_{1}\right)-X_{5}\left(t,\;\;T,\;\;C,\;\;D,\;\;IL_{2},\;\;Z_{2}\right){|}^{2}\leq |w^{\prime }{|}^{2}a^{2}|\left(Z_{1}-Z_{2}\right){|}^{2}, \end{array}$$

$$\begin{array}{c} \underset{t\in D_{Z}}{\text{sup}}\;|X_{5}\left(t,\;\;T,\;\;C,\;\;D,\;\;IL_{2},\;\;Z_{1}\right)-X_{5}\left(t,\;\;T,\;\;C,\;\;D,\;\;IL_{2},\;\;Z_{2}\right){|}^{2}\leq \\ \underset{t\in D_{w^{\prime }}}{\text{sup}}\;|w^{\prime }{|}^{2}a^{2}\;\underset{t\in D_{Z}}{\text{sup}}\;|Z_{1}-Z_{2}{|}^{2}, \end{array}$$

$$\begin{array}{c} \|X_{5}\left(t,\;\;T,\;\;C,\;\;D,\;\;IL_{2},\;\;Z_{1}\right)-X_{5}\left(t,\;\;T,\;\;C,\;\;D,\;\;IL_{2},\;\;Z_{2}\right){\|}_{\infty }^{2}\leq \|w^{\prime }{\|}_{\infty }^{2}a^{2}\|Z_{1}-Z_{2}{\|}_{\infty }^{2}, \end{array}$$

$$\begin{array}{c} \|X_{5}\left(t,\;\;T,\;\;C,\;\;D,\;\;IL_{2},\;\;Z_{1}\right)-X_{5}\left(t,\;\;T,\;\;C,\;\;D,\;\;IL_{2},\;\;Z_{2}\right){\|}_{\infty }^{2}\leq \bar{K_{5}}\|Z_{1}-Z_{2}{\|}_{\infty }^{2}, \end{array}$$

involving

$$\begin{array}{c} \bar{K_{5}}=\|w^{\prime }{\|}_{\infty }^{2}a^{2}. \end{array}$$

The system (3) therefore has a unique solution under the following condition.

$$\begin{array}{c} \text{max}\{\begin{array}{c} \frac{3}{\alpha^{2}\|T{\|}_{\infty }^{2}}(\alpha^{2}\beta^{2}\|T{\|}_{\infty }^{2}+\gamma^{2}+\phi^{2}\|C{\|}_{\infty }^{2}),\\ \frac{2(\phi^{2}\|T{\|}_{\infty }^{2}+\rho^{2}\|D{\|}_{\infty }^{2})}{\kappa^{2}\|C{\|}_{\infty }^{2}},\\ \frac{3}{\mu^{2}}(\rho^{2}\|C{\|}_{\infty }^{2}+\omega^{2}+\lambda^{2}),\\ \frac{d^{2}}{\lambda^{2}\|D{\|}_{\infty }^{2}},\\ \frac{a^{2}}{d^{2}\|IL_{2}{\|}_{\infty }^{2}}, \end{array}<1 \end{array}$$

## 5 Global stability for developed system

We use Lyapunov’s approach and LaSalle’s concept of invariance to analyze global stability and determine the conditions for eliminating diseases.

### 5.1 Lyapunov’s first derivative

**Theorem 5.1** Endemic equilibrium of the lung cancer model are globally asymptotically stable, if reproductive number *R*_0_> 1.

**Proof:** Lyapunov function can be expressed in the following manner.

$$\begin{array}{c} L\left(T^{*},C^{*},D^{*},IL_{2}^{*},Z^{*}\right)=(T-T^{*}-T^{*}\;\text{log}\;\frac{T}{T^{*}})+(C-C^{*}-C^{*}\;\text{log}\;\frac{C}{C^{*}})+\\ (D-D^{*}-D^{*}\;\text{log}\;\frac{D}{D^{*}})+(IL_{2}-IL_{2}^{*}-IL_{2}^{*}\;\text{log}\;\frac{IL_{2}}{IL_{2}^{*}})+(Z-Z^{*}-Z^{*}\;\text{log}\;\frac{Z}{Z^{*}}). \end{array}$$

We achieve by applying a derivative on both sides

$$\begin{array}{c} \begin{array}{c} FFM\\ 0 \end{array}D_{t}^{\xi ,\lambda }L=(\frac{T-T^{*}}{T})\begin{array}{c} FFM\\ 0 \end{array}D_{t}^{\xi ,\lambda }T+(\frac{C-C^{*}}{C})\begin{array}{c} FFM\\ 0 \end{array}D_{t}^{\xi ,\lambda }C+\\ (\frac{D-D^{*}}{D})\begin{array}{c} FFM\\ 0 \end{array}D_{t}^{\xi ,\lambda }D+(\frac{IL_{2}-IL_{2}^{*}}{IL_{2}})\begin{array}{c} FFM\\ 0 \end{array}D_{t}^{\xi ,\lambda }IL_{2}+(\frac{Z-Z^{*}}{Z})\begin{array}{c} FFM\\ 0 \end{array}D_{t}^{\xi ,\lambda }Z. \end{array}$$

We get,

$$\begin{array}{c} \begin{array}{c} FFM\\ 0 \end{array}D_{t}^{\xi ,\lambda }L=(\frac{T-T^{*}}{T})(\alpha T(1-\beta T)-\gamma T-\phi CT)+(\frac{C-C^{*}}{C})(\phi CT+\rho CD-\kappa C)+(\frac{D-D^{*}}{D})\\ \times (\mu -\rho CD-\omega D-\lambda D)+(\frac{IL_{2}-IL_{2}^{*}}{IL_{2}})(\lambda D-dIL_{2})+(\frac{Z-Z^{*}}{Z})(dIL_{2}-aZ). \end{array}$$

placing $T=T-T^{*},C=C-C^{*},D=D-D^{*},IL_{2}=IL_{2}-IL_{2}^{*},Z=Z-Z^{*}$ results in

$$\begin{array}{c} \begin{array}{c} FFM\\ 0 \end{array}D_{t}^{\xi ,\lambda }L=\alpha \frac{{\left(T-T^{*}\right)}^{2}}{T}-\alpha \beta \frac{{\left(T-T^{*}\right)}^{3}}{T}-\gamma \frac{{\left(T-T^{*}\right)}^{2}}{T}\\ -\phi C\frac{{\left(T-T^{*}\right)}^{2}}{T}+\phi C^{*}\frac{{\left(T-T^{*}\right)}^{2}}{T}+\phi T\frac{{\left(C-C^{*}\right)}^{2}}{C} \end{array}$$

$$\begin{array}{c} -\phi T^{*}\frac{{\left(C-C^{*}\right)}^{2}}{C}+\rho D\frac{{\left(C-C^{*}\right)}^{2}}{C}-\rho D^{*}\frac{{\left(C-C^{*}\right)}^{2}}{C}\\ -\kappa \frac{{\left(C-C^{*}\right)}^{2}}{C}+\mu -\mu \frac{D^{*}}{D}-\rho C\frac{{\left(D-D^{*}\right)}^{2}}{D} \end{array}$$

$$\begin{array}{c} +\rho C^{*}\frac{{\left(D-D^{*}\right)}^{2}}{D}-\omega \frac{{\left(D-D^{*}\right)}^{2}}{D}-\lambda \frac{{\left(D-D^{*}\right)}^{2}}{D}+\lambda D\\ -\lambda D\frac{IL_{2}^{*}}{IL_{2}}-\lambda D^{*}+\lambda D^{*}\frac{IL_{2}^{*}}{IL_{2}}-d\frac{{\left(IL_{2}-IL_{2}^{*}\right)}^{2}}{IL_{2}}. \end{array}$$

we can write

$$\begin{array}{c} FFM\\ 0 \end{array}D_{t}^{\xi ,\lambda }L=\Sigma -\Omega .$$

where

$$\begin{array}{c} \Sigma =\alpha \frac{{\left(T-T^{*}\right)}^{2}}{T}+\phi C^{*}\frac{{\left(T-T^{*}\right)}^{2}}{T}+\phi T\frac{{\left(C-C^{*}\right)}^{2}}{C}\\ +\rho D\frac{{\left(C-C^{*}\right)}^{2}}{C}+\mu +\rho C^{*}\frac{{\left(D-D^{*}\right)}^{2}}{D}+\lambda D+\lambda D^{*}\frac{IL_{2}^{*}}{IL_{2}}. \end{array}$$

and

$$\begin{array}{c} \Omega =\alpha \beta \frac{{\left(T-T^{*}\right)}^{3}}{T}+\gamma \frac{{\left(T-T^{*}\right)}^{2}}{T}+\phi C\frac{{\left(T-T^{*}\right)}^{2}}{T}+\phi T^{*}\frac{{\left(C-C^{*}\right)}^{2}}{C}\\ +\rho D^{*}\frac{{\left(C-C^{*}\right)}^{2}}{C}+\kappa \frac{{\left(C-C^{*}\right)}^{2}}{C}+\mu \frac{D^{*}}{D}+\rho C\frac{{\left(D-D^{*}\right)}^{2}}{D}\\ +\omega \frac{{\left(D-D^{*}\right)}^{2}}{D}+\lambda \frac{{\left(D-D^{*}\right)}^{2}}{D}+\lambda D\frac{IL_{2}^{*}}{IL_{2}}+\lambda D^{*}+d\frac{{\left(IL_{2}-IL_{2}^{*}\right)}^{2}}{IL_{2}}. \end{array}$$

We conclude that if Σ < Ω, this yields $\begin{array}{c} FFM\\ 0 \end{array}D_{t}^{\xi ,\lambda }L<0$, however when $T=T^{*},C=C^{*},D=D^{*},IL_{2}=IL_{2}^{*},Z=Z^{*}$.

$\Sigma -\Omega =0\;\Rightarrow \begin{array}{c} FFM\\ 0 \end{array}D_{t}^{\xi ,\lambda }L=0$ .

We can observe that $\left\{\left(T^{*},C^{*},D^{*},IL_{2}^{*},Z^{*}\right)\in \Gamma :\begin{array}{c} FFM\\ 0 \end{array}D_{t}^{\xi ,\lambda }L=0\right\}$ represents the point *D*_2_ for developed model.

According to Lasalles’ concept of invariance, the *D*_2_ is globally uniformly stable in Γ if Σ < Ω.

## 6 Solutions by fractal fractional operator

Now, we will develop a solution using numerical approach for our newly developed model given in Eq (3). We use ML kernel in the current scenario instead of classical derivative operator. Furthermore, we will use the variable order version.

$$\begin{array}{ccc} \begin{array}{c} FFM\\ 0 \end{array}D_{t}^{\xi ,\lambda }T\left(t\right) & = & \alpha T(1-\beta T)-\gamma T-\phi CT,\\ \begin{array}{c} FFM\\ 0 \end{array}D_{t}^{\xi ,\lambda }C\left(t\right) & = & \phi CT+\rho CD-\kappa C,\\ \begin{array}{c} FFM\\ 0 \end{array}D_{t}^{\xi ,\lambda }D\left(t\right) & = & \mu -\rho CD-\omega D-\lambda D,\\ \begin{array}{c} FFM\\ 0 \end{array}D_{t}^{\xi ,\lambda }IL_{2}\left(t\right) & = & \lambda D-dIL_{2},\\ \begin{array}{c} FFM\\ 0 \end{array}D_{t}^{\xi ,\lambda }Z\left(t\right) & = & dIL_{2}-aZ. \end{array}$$

For clarity, we express the above equation as follows:

$$\begin{array}{ccc} \begin{array}{c} FFM\\ 0 \end{array}D_{t}^{\xi ,\lambda }T\left(t\right) & = & T_{1}\left(t,T,G\right),\\ \begin{array}{c} FFM\\ 0 \end{array}D_{t}^{\xi ,\lambda }C\left(t\right) & = & C_{1}\left(t,T,G\right),\\ \begin{array}{c} FFM\\ 0 \end{array}D_{t}^{\xi ,\lambda }D\left(t\right) & = & D_{1}\left(t,T,G\right),\\ \begin{array}{c} FFM\\ 0 \end{array}D_{t}^{\xi ,\lambda }IL_{2}\left(t\right) & = & {IL_{2}}_{1}\left(t,T,G\right),\\ \begin{array}{c} FFM\\ 0 \end{array}D_{t}^{\xi ,\lambda }Z\left(t\right) & = & Z_{1}\left(t,T,G\right). \end{array}$$

Involving

$$\begin{array}{ccc} T_{1}\left(\;t,\;T,\;G\right) & = & \;\alpha \;T(\;1\;-\;\beta \;T)-\gamma T-\phi CT,\\ C_{1}\left(\;t,\;T,\;G\right) & = & \phi CT+\rho CD-\kappa C,\\ D_{1}\left(t,T,G\right) & = & \mu -\rho CD-\omega D-\lambda D,\\ {IL_{2}}_{1}\left(t,S,G\right) & = & \lambda D-dIL_{2},\\ Z_{1}\left(t,T,G\right) & = & dIL_{2}-aZ. \end{array}$$

After using the fractal-fractional integral with the ML kernel, we obtain the following results.

$$\begin{array}{ccc} T(t_{\eta }+1) & = & \frac{\lambda (1-\xi )}{AB(\xi )}t_{\eta }^{\lambda -1}T_{1}\left(t_{\eta },T\left(t_{\eta }\right),G\left(t_{\eta }\right)\right)\\ & + & \frac{\xi \lambda }{AB(\xi )\Gamma (\xi )}\;\sum_{\nu =2}^{\eta }\;\int_{t_{\nu }}^{t_{\nu +1}}T_{1}\left(t,T,G\right)\tau^{\xi -1}{\left(t_{\eta +1}-\tau \right)}^{\xi -1}d\tau ,\\ C(t_{\eta }+1) & = & \frac{\lambda (1-\xi )}{AB(\xi )}t_{\eta }^{\lambda -1}C_{1}\left(t_{\eta },T\left(t_{\eta }\right),G\left(t_{\eta }\right)\right)\\ & + & \frac{\xi \lambda }{AB(\xi )\Gamma (\xi )}\;\sum_{\nu =2}^{\eta }\;\int_{t_{\nu }}^{t_{\nu +1}}C_{1}\left(t,T,G\right)\tau^{\xi -1}{\left(t_{\eta +1}-\tau \right)}^{\xi -1}d\tau ,\\ D(t_{\eta }+1) & = & \frac{\lambda (1-\xi )}{AB(\xi )}t_{\eta }^{\lambda -1}D_{1}\left(t_{\eta },T\left(t_{\eta }\right),G\left(t_{\eta }\right)\right)\\ & + & \frac{\xi \lambda }{AB(\xi )\Gamma (\xi )}\;\sum_{\nu =2}^{\eta }\;\int_{t_{\nu }}^{t_{\nu +1}}D_{1}\left(t,T,G\right)\tau^{\xi -1}{\left(t_{\eta +1}-\tau \right)}^{\xi -1}d\tau ,\\ IL_{2}\left(t_{\eta }+1\right) & = & \frac{\lambda (1-\xi )}{AB(\xi )}t_{\eta }^{\lambda -1}{IL_{2}}_{1}\left(t_{\eta },T\left(t_{\eta }\right),G\left(t_{\eta }\right)\right)\\ & + & \frac{\xi \lambda }{AB(\xi )\Gamma (\xi )}\;\sum_{\nu =2}^{\eta }\;\int_{t_{\nu }}^{t_{\nu +1}}{IL_{2}}_{1}\left(t,T,G\right)\tau^{\xi -1}{\left(t_{\eta +1}-\tau \right)}^{\xi -1}d\tau ,\\ Z(t_{\eta }+1) & = & \frac{\lambda (1-\xi )}{AB(\xi )}t_{\eta }^{\lambda -1}Z_{1}\left(t_{\eta },T\left(t_{\eta }\right),G\left(t_{\eta }\right)\right)\\ & + & \frac{\xi \lambda }{AB(\xi )\Gamma (\xi )}\;\sum_{\nu =2}^{\eta }\;\int_{t_{\nu }}^{t_{\nu +1}}Z_{1}\left(t,T,G\right)\tau^{\xi -1}{\left(t_{\eta +1}-\tau \right)}^{\xi -1}d\tau . \end{array}$$ (5)

where *G*(*t*_*η*_) = *C*(*t*_*η*_), *D*(*t*_*η*_), *IL*_2_(*t*_*η*_), *Z*(*t*_*η*_).

Remember that the Newton polynomial can be obtained by using the Newton interpolation formula.

$$\begin{array}{ccc} N(t,T,G) & ≃ & N\left(t_{\eta -2},T_{\eta -2},G_{\eta -2}\right)+\frac{1}{\Delta t}[N\left(t_{\eta -1},T_{\eta -1},G_{\eta -1}\right)\\ & - & N\left(t_{\eta -2},T_{\eta -2},G_{\eta -2}\right)](\tau -t_{\eta -2})+\frac{1}{2\Delta t^{2}}[N\left(t_{\eta },T_{\eta },C_{\eta },D_{\eta },{IL_{2}}_{\eta },Z_{\eta }\right)\\ & - & 2N\left(t_{\eta -1},T_{\eta -1},G_{\eta -1}\right)-N\left(t_{\eta -2},T_{\eta -2},G_{\eta -2}\right)](\tau -t_{\eta -2})(\tau -t_{\eta -1}). \end{array}$$

where *G*_*η*−2_ = *C*_*η*−2_, *D*_*η*−2_, *IL*_2__*η*−2_, *Z*_*η*−2_, *G*_*η*−1_ = *C*_*η*−1_, *D*_*η*−1_, *IL*_2__*η*−1_, *Z*_*η*−1_.

When we substitute the Newton polynomial into system of Eq (5), we obtain the following.

$$\begin{array}{c} T^{\eta +1}=\frac{\lambda (1-\xi )}{AB(\xi )}t_{\eta }^{\lambda -1}T_{1}\left(t_{\eta },T\left(t_{\eta }\right),G\left(t_{\eta }\right)\right)+\frac{\xi \lambda }{AB(\xi )\Gamma (\xi )}\sum_{\nu =2}^{\eta }S_{1}\left(t_{\nu -2},T^{\nu -2},G^{\nu -2}\right) \end{array}$$

$$\begin{array}{c} \times t_{\nu -2}^{\lambda -1}\int_{t_{\nu }}^{t_{\nu +1}}{\left(t_{\eta +1}-\tau \right)}^{\xi -1}d\tau +\frac{\xi \lambda }{AB(\xi )\Gamma (\xi )}\sum_{\nu =2}^{\eta }\frac{1}{\Delta t}[t_{\nu -1}^{\lambda -1}T_{1}\left(t_{\nu -1},T^{\nu -1},G^{\nu -1}\right) \end{array}$$

$$\begin{array}{c} -t_{\nu -2}^{\lambda -1}T_{1}\left(t_{\nu -2},T^{\nu -2},G^{\nu -2}\right)]\int_{t_{\nu }}^{t_{\nu +1}}\left(\tau -t_{\nu -2}\right){\left(t_{\eta +1}-\tau \right)}^{\xi -1}d\tau \end{array}$$

$$\begin{array}{c} +\frac{\xi }{AB(\xi )\Gamma (\xi )}\sum_{\nu =2}^{\eta }\frac{1}{2\Delta t^{2}}[t_{\nu }^{\lambda -1}T_{1}\left(t_{\nu },T^{\nu },G^{\nu }\right)-2t_{\nu -1}^{\lambda -1}T_{1}\left(t_{\nu -1},T^{\nu -1},G^{\nu -1}\right)\\ +t_{\nu -2}^{\lambda -1}T_{1}\left(t_{\nu -2},T^{\nu -2},G^{\nu -2}\right)] \end{array}$$

$$\begin{array}{c} \times \int_{t_{\nu }}^{t_{\nu +1}}\left(\;\tau \;-\;t_{\;\nu \;-\;2}\right)\left(\;\tau \;-\;t_{\;\nu \;-\;1}\right){\left(t_{\eta +1}-\tau \right)}^{\xi -1}d\tau , \end{array}$$

$$\begin{array}{c} C^{\eta +1}=\frac{\lambda (1-\xi )}{AB(\xi )}t_{\eta }^{\lambda -1}C_{1}\left(t_{\eta },T\left(t_{\eta }\right),G\left(t_{\eta }\right)\right)+\frac{\xi \lambda }{AB(\xi )\Gamma (\xi )}\sum_{\nu =2}^{\eta }C_{1}\left(t_{\nu -2},T^{\nu -2},G^{\nu -2}\right) \end{array}$$

$$\begin{array}{c} \times t_{\nu -2}^{\lambda -1}\int_{t_{\nu }}^{t_{\nu +1}}{\left(t_{\eta +1}-\tau \right)}^{\xi -1}d\tau \;+\frac{\xi \lambda }{AB(\xi )\Gamma (\xi )}\sum_{\nu =2}^{\eta }\frac{1}{\Delta t}[t_{\nu -1}^{\lambda -1}C_{1}\left(t_{\nu -1},T^{\nu -1},G^{\nu -1}\right) \end{array}$$

$$\begin{array}{c} -t_{\nu -2}^{\lambda -1}C_{1}\left(t_{\nu -2},T^{\nu -2},G^{\nu -2}\right)]\int_{t_{\nu }}^{t_{\nu +1}}\left(\tau -t_{\nu -2}\right){\left(t_{\eta +1}-\tau \right)}^{\xi -1}d\tau \end{array}$$

$$\begin{array}{c} +\frac{\xi \lambda }{AB(\xi )\Gamma (\xi )}\sum_{\nu =2}^{\eta }\frac{1}{2\Delta t^{2}}[t_{\nu }^{\lambda -1}C_{1}\left(t_{\nu },T^{\nu },G^{\nu }\right)-2t_{\nu -1}^{\lambda -1}C_{1}\left(t_{\nu -1},T^{\nu -1},G^{\nu -1}\right)\\ +t_{\nu -2}^{\lambda -1}C_{1}\left(t_{\nu -2},T^{\nu -2},G^{\nu -2}\right)] \end{array}$$

$$\begin{array}{c} \times \int_{t_{\nu }}^{t_{\nu +1}}\left(\;\tau \;-\;t_{\;\nu \;-\;2}\right)\left(\;\tau \;-\;t_{\;\nu \;-\;1}\right){\left(t_{\eta +1}-\tau \right)}^{\xi -1}d\tau , \end{array}$$

$$\begin{array}{c} D^{\eta +1}=\frac{\lambda (1-\xi )}{AB(\xi )}t_{\eta }^{\lambda -1}I_{1}\left(t_{\eta },T\left(t_{\eta }\right),G\left(t_{\eta }\right)\right)+\frac{\xi \lambda }{AB(\xi )\Gamma (\xi )}\sum_{\nu =2}^{\eta }D_{1}\left(t_{\nu -2},T^{\nu -2},G^{\nu -2}\right) \end{array}$$

$$\begin{array}{c} \times t_{\nu -2}^{\lambda -1}\int_{t_{\nu }}^{t_{\nu +1}}{\left(t_{\eta +1}-\tau \right)}^{\xi -1}d\tau +\frac{\xi \lambda }{AB(\xi )\Gamma (\xi )}\sum_{\nu =2}^{\eta }\frac{1}{\Delta t}[t_{\nu -1}^{\lambda -1}D_{1}\left(t_{\nu -1},T^{\nu -1},G^{\nu -1}\right) \end{array}$$

$$\begin{array}{c} -t_{\nu -2}^{\lambda -1}D_{1}\left(t_{\nu -2},T^{\nu -2},G^{\nu -2}\right)]\int_{t_{\nu }}^{t_{\nu +1}}\left(\tau -t_{\nu -2}\right){\left(t_{\eta +1}-\tau \right)}^{\xi -1}d\tau \end{array}$$

$$\begin{array}{c} +\frac{\xi \lambda }{AB(\xi )\Gamma (\xi )}\sum_{\nu =2}^{\eta }\frac{1}{2\Delta t^{2}}[t_{\nu }^{\lambda -1}D_{1}\left(t_{\nu },T^{\nu },G^{\nu }\right)-2t_{\nu -1}^{\lambda -1}D_{1}\left(t_{\nu -1},T^{\nu -1},G^{\nu -1}\right)\\ +t_{\nu -2}^{\lambda -1}D_{1}\left(t_{\nu -2},T^{\nu -2},G^{\nu -2}\right)] \end{array}$$

$$\begin{array}{c} \times \int_{t_{\nu }}^{t_{\nu +1}}\left(\;\tau \;-\;t_{\;\nu \;-\;2}\right)\left(\;\tau \;-\;t_{\;\nu \;-\;1}\right){\left(t_{\eta +1}-\tau \right)}^{\xi -1}d\tau , \end{array}$$ (6)

$$\begin{array}{c} IL_{2}^{\eta +1}=\frac{\lambda (1-\xi )}{AB(\xi )}t_{\eta }^{\lambda -1}{IL_{2}}_{1}\left(t_{\eta },T\left(t_{\eta }\right),G\left(t_{\eta }\right)\right)+\frac{\xi \lambda }{AB(\xi )\Gamma (\xi )}\sum_{\nu =2}^{\eta }{IL_{2}}_{1}\left(t_{\nu -2},T^{\nu -2},G^{\nu -2}\right) \end{array}$$

$$\begin{array}{c} \times t_{\nu -2}^{\lambda -1}\int_{t_{\nu }}^{t_{\nu +1}}{\left(t_{\eta +1}-\tau \right)}^{\xi -1}d\tau +\frac{\xi \lambda }{AB(\xi )\Gamma (\xi )}\sum_{\nu =2}^{\eta }\frac{1}{\Delta t}[t_{\nu -1}^{\lambda -1}{IL_{2}}_{1}\left(t_{\nu -1},T^{\nu -1},G^{\nu -1}\right) \end{array}$$

$$\begin{array}{c} -t_{\nu -2}^{\lambda -1}{IL_{2}}_{1}\left(t_{\nu -2},T^{\nu -2},G^{\nu -2}\right)]\int_{t_{\nu }}^{t_{\nu +1}}\left(\tau -t_{\nu -2}\right){\left(t_{\eta +1}-\tau \right)}^{\xi -1}d\tau \end{array}$$

$$\begin{array}{c} +\frac{\xi \lambda }{AB(\xi )\Gamma (\xi )}\sum_{\nu =2}^{\eta }\frac{1}{2\Delta t^{2}}[t_{\nu }^{\lambda -1}{IL_{2}}_{1}\left(t_{\nu },T^{\nu },G^{\nu }\right)-2t_{\nu -1}^{\lambda -1}{IL_{2}}_{1}\left(t_{\nu -1},T^{\nu -1},G^{\nu -1}\right)\\ +t_{\nu -2}^{\lambda -1}{IL_{2}}_{1}\left(t_{\nu -2},T^{\nu -2},G^{\nu -2}\right)] \end{array}$$

$$\begin{array}{c} \times \int_{t_{\nu }}^{t_{\nu +1}}\left(\;\tau \;-\;t_{\;\nu \;-\;2}\right)\left(\;\tau \;-\;t_{\;\nu \;-\;1}\right){\left(t_{\eta +1}-\tau \right)}^{\xi -1}d\tau , \end{array}$$

$$\begin{array}{c} Z^{\eta +1}=\frac{\lambda (1-\xi )}{AB(\xi )}t_{\eta }^{\lambda -1}Z_{1}\left(t_{\eta },T\left(t_{\eta }\right),G\left(t_{\eta }\right)\right)+\frac{\xi \lambda }{AB(\xi )\Gamma (\xi )}\sum_{\nu =2}^{\eta }Z_{1}\left(t_{\nu -2},T^{\nu -2},G^{\nu -2}\right) \end{array}$$

$$\begin{array}{c} \times t_{\nu -2}^{\lambda -1}\int_{t_{\nu }}^{t_{\nu +1}}{\left(t_{\eta +1}-\tau \right)}^{\xi -1}d\tau \;+\frac{\xi \lambda }{AB(\xi )\Gamma (\xi )}\sum_{\nu =2}^{\eta }\frac{1}{\Delta t}[t_{\nu -1}^{\lambda -1}Z_{1}\left(t_{\nu -1},T^{\nu -1},G^{\nu -1}\right) \end{array}$$

$$\begin{array}{c} -t_{\nu -2}^{\lambda -1}Z_{1}\left(t_{\nu -2},T^{\nu -2},G^{\nu -2}\right)]\int_{t_{\nu }}^{t_{\nu +1}}\left(\tau -t_{\nu -2}\right){\left(t_{\eta +1}-\tau \right)}^{\xi -1}d\tau \end{array}$$

$$\begin{array}{c} +\frac{\xi \lambda }{AB(\xi )\Gamma (\xi )}\sum_{\nu =2}^{\eta }\frac{1}{2\Delta t^{2}}[t_{\nu }^{\lambda -1}Z_{1}\left(t_{\nu },T^{\nu },G^{\nu }\right)-2t_{\nu -1}^{\lambda -1}Z_{1}\left(t_{\nu -1},T^{\nu -1},G^{\nu -1}\right)\\ +t_{\nu -2}^{\lambda -1}Z_{1}\left(t_{\nu -2},T^{\nu -2},G^{\nu -2}\right)] \end{array}$$

$$\begin{array}{c} \times \int_{t_{\nu }}^{t_{\nu +1}}\left(\;\tau \;-\;t_{\;\nu \;-\;2}\right)\left(\;\tau \;-\;t_{\;\nu \;-\;1}\right){\left(t_{\eta +1}-\tau \right)}^{\xi -1}d\tau . \end{array}$$

where $G^{\nu -2}=C^{\nu -2},D^{\nu -2},IL_{2}^{\nu -2},Z^{\nu -2}$, $G^{\nu -1}=C^{\nu -1},D^{\nu -1},IL_{2}^{\nu -1},Z^{\nu -1}$, $G^{\nu }=C^{\nu },D^{\nu },IL_{2}^{\nu },Z^{\nu }$, *G*(*t*_*η*_) = *C*(*t*_*η*_), *D*(*t*_*η*_), *IL*_2_(*t*_*η*_), *Z*(*t*_*η*_).

We can perform the following calculations for the integral in Eq (6).

$$\begin{array}{c} \int_{t_{\nu }}^{t_{\nu +1}}{\left(t_{\eta +1}-\tau \right)}^{\xi -1}d\tau =\frac{{\left(\Delta t\right)}^{\xi }}{\xi }[{\left(\eta -\nu +1\right)}^{\xi }-{\left(\eta -\nu \right)}^{\xi }],\\ \int_{t_{\nu }}^{t_{\nu +1}}\left(\tau -t_{\nu -2}\right){\left(t_{\eta +1}-\tau \right)}^{\xi -1}d\tau =\frac{{\left(\Delta t\right)}^{\xi +1}}{\xi (\xi +1)}[{\left(\eta -\nu +1\right)}^{\xi }\left(\eta -\nu +3+2\xi \right)\\ -{\left(\eta -\nu \right)}^{\xi }\left(\eta -\nu +3+3\xi \right)], \end{array}$$ (7)

$$\begin{array}{c} \int_{t_{\nu }}^{t_{\nu +1}}\left(\;\tau \;-\;t_{\;\nu \;-\;2}\right)\left(\;\tau \;-\;t_{\;\nu \;-\;1}\right){\left(t_{\eta +1}-\tau \right)}^{\xi -1}d\tau =\frac{{\left(\Delta t\right)}^{\xi +2}}{\xi (\xi +1)(\xi +2)}\\ {}[{\left(\eta -\nu +1\right)}^{\xi }\{2{\left(\eta -\nu \right)}^{2}+\left(3\xi +10\right)\left(\eta -\nu \right)+2\xi^{2}+9\xi +12\}-\\ {\left(\eta -\nu \right)}^{\xi }\{2{\left(\eta -\nu \right)}^{2}+\left(5\xi +10\right)\left(\eta -\nu \right)+6\xi^{2}+18\xi +12\}]. \end{array}$$

putting above all integral calculation values from Eq (7) into Eq (6).

We acquire the numerical solutions *T*(*t*), *C*(*t*), *D*(*t*), *IL*_2_(*t*) *and Z*(*t*).

$$\begin{array}{c} T^{\eta +1}=\frac{\lambda (1-\xi )}{AB(\xi )}t_{\eta }^{\lambda -1}T_{1}\left(t_{\eta },T\left(t_{\eta }\right),G\left(t_{\eta }\right)\right)+\frac{\xi \lambda {\left(\Delta t\right)}^{\xi }}{AB(\xi )\Gamma (\xi +1)}\sum_{\nu =2}^{\eta }T_{1}\left(t_{\nu -2},T^{\nu -2},G^{\nu -2}\right) \end{array}$$

$$\begin{array}{c} \times t_{\nu -2}^{\lambda -1}[{\left(\;\eta \;-\;\nu \;+\;1\right)}^{\xi }-{\left(\eta -\nu \right)}^{\xi }]+\frac{\xi \lambda {\left(\Delta t\right)}^{\xi }}{AB(\xi )\Gamma (\xi +2)}\sum_{\nu =2}^{\eta }[t_{\nu -1}^{\lambda -1}T_{1}\left(t_{\nu -1},T^{\nu -1},G^{\nu -1}\right) \end{array}$$

$$\begin{array}{c} -t_{\nu -2}^{\lambda -1}T_{1}\left(t_{\nu -2},T^{\nu -2},G^{\nu -2}\right)][{\left(\;\eta \;-\;\nu \;+\;1\right)}^{\xi }\left(\eta -\nu +3+2\xi \right)-{\left(\eta -\nu \right)}^{\xi }\left(\eta -\nu +3+3\xi \right)] \end{array}$$

$$\begin{array}{c} +\frac{\xi \lambda {\left(\Delta t\right)}^{\xi }}{2AB(\xi )\Gamma (\xi +3)}\sum_{\nu =2}^{\eta }[t_{\nu }^{\lambda -1}T_{1}\left(t_{\nu },T^{\nu },G^{\nu }\right)-2t_{\nu -1}^{\lambda -1}T_{1}\left(t_{\nu -1},T^{\nu -1},G^{\nu -1}\right) \end{array}$$

$$\begin{array}{c} +t_{\nu -2}^{\lambda -1}T_{1}\left(t_{\nu -2},T^{\nu -2},G^{\nu -2}\right)][{\left(\;\eta \;-\;\nu \;+\;1\right)}^{\xi }\{2{\left(\eta -\nu \right)}^{2}+\left(3\xi +10\right)\left(\eta -\nu \right)+2\xi^{2}+9\xi +12\} \end{array}$$

$$\begin{array}{c} -{\left(\eta -\nu \right)}^{\xi }\times \{2{\left(\eta -\nu \right)}^{2}+\left(5\xi +10\right)\left(\eta -\nu \right)+6\xi^{2}+18\xi +12\}], \end{array}$$

$$\begin{array}{c} C^{\eta +1}=\frac{\lambda (1-\xi )}{AB(\xi )}t_{\eta }^{\lambda -1}C_{1}\left(t_{\eta },T\left(t_{\eta }\right),G\left(t_{\eta }\right)\right)+\frac{\xi \lambda {\left(\Delta t\right)}^{\xi }}{AB(\xi )\Gamma (\xi +1)}\sum_{\nu =2}^{\eta }C_{1}\left(t_{\nu -2},T^{\nu -2},G^{\nu -2}\right) \end{array}$$

$$\begin{array}{c} \times t_{\nu -2}^{\lambda -1}[{\left(\;\eta \;-\;\nu \;+\;1\right)}^{\xi }-{\left(\eta -\nu \right)}^{\xi }]+\frac{\xi \lambda {\left(\Delta t\right)}^{\xi }}{AB(\xi )\Gamma (\xi +2)}\sum_{\nu =2}^{\eta }[t_{\nu -1}^{\lambda -1}C_{1}\left(t_{\nu -1},T^{\nu -1},G^{\nu -1}\right) \end{array}$$

$$\begin{array}{c} -t_{\nu -2}^{\lambda -1}C_{1}\left(t_{\nu -2},T^{\nu -2},G^{\nu -2}\right)][{\left(\;\eta \;-\;\nu \;+\;1\right)}^{\xi }\left(\eta -\nu +3+2\xi \right)-{\left(\eta -\nu \right)}^{\xi }\left(\eta -\nu +3+3\xi \right)] \end{array}$$

$$\begin{array}{c} +\frac{\xi \lambda {\left(\Delta t\right)}^{\xi }}{2AB(\xi )\Gamma (\xi +3)}\sum_{\nu =2}^{\eta }[t_{\nu }^{\lambda -1}C_{1}\left(t_{\nu },T^{\nu },G^{\nu }\right)-2t_{\nu -1}^{\lambda -1}C_{1}\left(t_{\nu -1},T^{\nu -1},G^{\nu -1}\right) \end{array}$$

$$\begin{array}{c} +t_{\nu -2}^{\lambda -1}C_{1}\left(t_{\nu -2},T^{\nu -2},G^{\nu -2}\right)][{\left(\;\eta \;-\;\nu \;+\;1\right)}^{\xi }\{2{\left(\eta -\nu \right)}^{2}+\left(3\xi +10\right)\left(\eta -\nu \right)+2\xi^{2}+9\xi +12\} \end{array}$$

$$\begin{array}{c} -{\left(\eta -\nu \right)}^{\xi }\times \{2{\left(\eta -\nu \right)}^{2}+\left(5\xi +10\right)\left(\eta -\nu \right)+6\xi^{2}+18\xi +12\}], \end{array}$$

$$\begin{array}{c} D^{\eta +1}=\frac{\lambda (1-\xi )}{AB(\xi )}t_{\eta }^{\lambda -1}D_{1}\left(t_{\eta },T\left(t_{\eta }\right),G\left(t_{\eta }\right)\right)+\frac{\xi \lambda {\left(\Delta t\right)}^{\xi }}{AB(\xi )\Gamma (\xi +1)}\sum_{\nu =2}^{\eta }D_{1}\left(t_{\nu -2},T^{\nu -2},G^{\nu -2}\right) \end{array}$$

$$\begin{array}{c} \times t_{\nu -2}^{\lambda -1}[{\left(\;\eta \;-\;\nu \;+\;1\right)}^{\xi }-{\left(\eta -\nu \right)}^{\xi }]+\frac{\xi \lambda {\left(\Delta t\right)}^{\xi }}{AB(\xi )\Gamma (\xi +2)}\sum_{\nu =2}^{\eta }[t_{\nu -1}^{\lambda -1}D_{1}\left(t_{\nu -1},T^{\nu -1},G^{\nu -1}\right) \end{array}$$

$$\begin{array}{c} -t_{\nu -2}^{\lambda -1}D_{1}\left(t_{\nu -2},T^{\nu -2},G^{\nu -2}\right)][{\left(\;\eta \;-\;\nu \;+\;1\right)}^{\xi }\left(\eta -\nu +3+2\xi \right)-{\left(\eta -\nu \right)}^{\xi }\left(\eta -\nu +3+3\xi \right)] \end{array}$$

$$\begin{array}{c} +\frac{\xi \lambda {\left(\Delta t\right)}^{\xi }}{2AB(\xi )\Gamma (\xi +3)}\sum_{\nu =2}^{\eta }[t_{\nu }^{\lambda -1}D_{1}\left(t_{\nu },T^{\nu },G^{\nu }\right)-2t_{\nu -1}^{\lambda -1}D_{1}\left(t_{\nu -1},T^{\nu -1},G^{\nu -1}\right) \end{array}$$

$$\begin{array}{c} +t_{\nu -2}^{\lambda -1}D_{1}\left(t_{\nu -2},T^{\nu -2},G^{\nu -2}\right)][{\left(\;\eta \;-\;\nu \;+\;1\right)}^{\xi }\{2{\left(\eta -\nu \right)}^{2}+\left(3\xi +10\right)\left(\eta -\nu \right)+2\xi^{2}+9\xi +12\} \end{array}$$

$$\begin{array}{c} -{\left(\eta -\nu \right)}^{\xi }\times \{2{\left(\eta -\nu \right)}^{2}+\left(5\xi +10\right)\left(\eta -\nu \right)+6\xi^{2}+18\xi +12\}], \end{array}$$

$$\begin{array}{c} IL_{2}^{\eta +1}=\frac{\lambda (1-\xi )}{AB(\xi )}t_{\eta }^{\lambda -1}{IL_{2}}_{1}\left(t_{\eta },T\left(t_{\eta }\right),G\left(t_{\eta }\right)\right)+\frac{\xi \lambda {\left(\Delta t\right)}^{\xi }}{AB(\xi )\Gamma (\xi +1)}\sum_{\nu =2}^{\eta }{IL_{2}}_{1}\left(t_{\nu -2},T^{\nu -2},G^{\nu -2}\right) \end{array}$$

$$\begin{array}{c} \times t_{\nu -2}^{\lambda -1}[{\left(\;\eta \;-\;\nu \;+\;1\right)}^{\xi }-{\left(\eta -\nu \right)}^{\xi }]+\frac{\xi \lambda {\left(\Delta t\right)}^{\xi }}{AB(\xi )\Gamma (\xi +2)}\sum_{\nu =2}^{\eta }[t_{\nu -1}^{\lambda -1}{IL_{2}}_{1}\left(t_{\nu -1},T^{\nu -1},G^{\nu -1}\right) \end{array}$$

$$\begin{array}{c} -t_{\nu -2}^{\lambda -1}{IL_{2}}_{1}\left(t_{\nu -2},T^{\nu -2},G^{\nu -2}\right)][{\left(\;\eta \;-\;\nu \;+\;1\right)}^{\xi }\left(\eta -\nu +3+2\xi \right)-{\left(\eta -\nu \right)}^{\xi }\left(\eta -\nu +3+3\xi \right)] \end{array}$$

$$\begin{array}{c} +\frac{\xi \lambda {\left(\Delta t\right)}^{\xi }}{2AB(\xi )\Gamma (\xi +3)}\sum_{\nu =2}^{\eta }[t_{\nu }^{\lambda -1}{IL_{2}}_{1}\left(t_{\nu },T^{\nu },G^{\nu }\right)-2t_{\nu -1}^{\lambda -1}{IL_{2}}_{1}\left(t_{\nu -1},T^{\nu -1},G^{\nu -1}\right) \end{array}$$

$$\begin{array}{c} +t_{\nu -2}^{\lambda -1}{IL_{2}}_{1}\left(t_{\nu -2},T^{\nu -2},G^{\nu -2}\right)][{\left(\;\eta \;-\;\nu \;+\;1\right)}^{\xi }\{2{\left(\eta -\nu \right)}^{2}+\left(3\xi +10\right)\left(\eta -\nu \right)+2\xi^{2}+9\xi +12\} \end{array}$$

$$\begin{array}{c} -{\left(\eta -\nu \right)}^{\xi }\times \{2{\left(\eta -\nu \right)}^{2}+\left(5\xi +10\right)\left(\eta -\nu \right)+6\xi^{2}+18\xi +12\}], \end{array}$$

$$\begin{array}{c} Z^{\eta +1}=\frac{\lambda (1-\xi )}{AB(\xi )}t_{\eta }^{\lambda -1}Z_{1}\left(t_{\eta },T\left(t_{\eta }\right),G\left(t_{\eta }\right)\right)+\frac{\xi \lambda {\left(\Delta t\right)}^{\xi }}{AB(\xi )\Gamma (\xi +1)}\sum_{\nu =2}^{\eta }Z_{1}\left(t_{\nu -2},T^{\nu -2},G^{\nu -2}\right) \end{array}$$

$$\begin{array}{c} \times t_{\nu -2}^{\lambda -1}[{\left(\;\eta \;-\;\nu \;+\;1\right)}^{\xi }-{\left(\eta -\nu \right)}^{\xi }]+\frac{\xi \lambda {\left(\Delta t\right)}^{\xi }}{AB(\xi )\Gamma (\xi +2)}\sum_{\nu =2}^{\eta }[t_{\nu -1}^{\lambda -1}Z_{1}\left(t_{\nu -1},T^{\nu -1},G^{\nu -1}\right) \end{array}$$

$$\begin{array}{c} -t_{\nu -2}^{\lambda -1}Z_{1}\left(t_{\nu -2},T^{\nu -2},G^{\nu -2}\right)][{\left(\;\eta \;-\;\nu \;+\;1\right)}^{\xi }\left(\eta -\nu +3+2\xi \right)-{\left(\eta -\nu \right)}^{\xi }\left(\eta -\nu +3+3\xi \right)] \end{array}$$

$$\begin{array}{c} +\frac{\xi \lambda {\left(\Delta t\right)}^{\xi }}{2AB(\xi )\Gamma (\xi +3)}\sum_{\nu =2}^{\eta }[t_{\nu }^{\lambda -1}Z_{1}\left(t_{\nu },T^{\nu },G^{\nu }\right)-2t_{\nu -1}^{\lambda -1}Z_{1}\left(t_{\nu -1},T^{\nu -1},G^{\nu -1}\right) \end{array}$$

$$\begin{array}{c} +t_{\nu -2}^{\lambda -1}Z_{1}\left(t_{\nu -2},T^{\nu -2},G^{\nu -2}\right)][{\left(\;\eta \;-\;\nu \;+\;1\right)}^{\xi }\{2{\left(\eta -\nu \right)}^{2}+\left(3\xi +10\right)\left(\eta -\nu \right)+2\xi^{2}+9\xi +12\} \end{array}$$

$$\begin{array}{c} -{\left(\eta -\nu \right)}^{\xi }\times \{2{\left(\eta -\nu \right)}^{2}+\left(5\xi +10\right)\left(\eta -\nu \right)+6\xi^{2}+18\xi +12\}]. \end{array}$$

## 7 Simulation explanation

**Fig 2:**
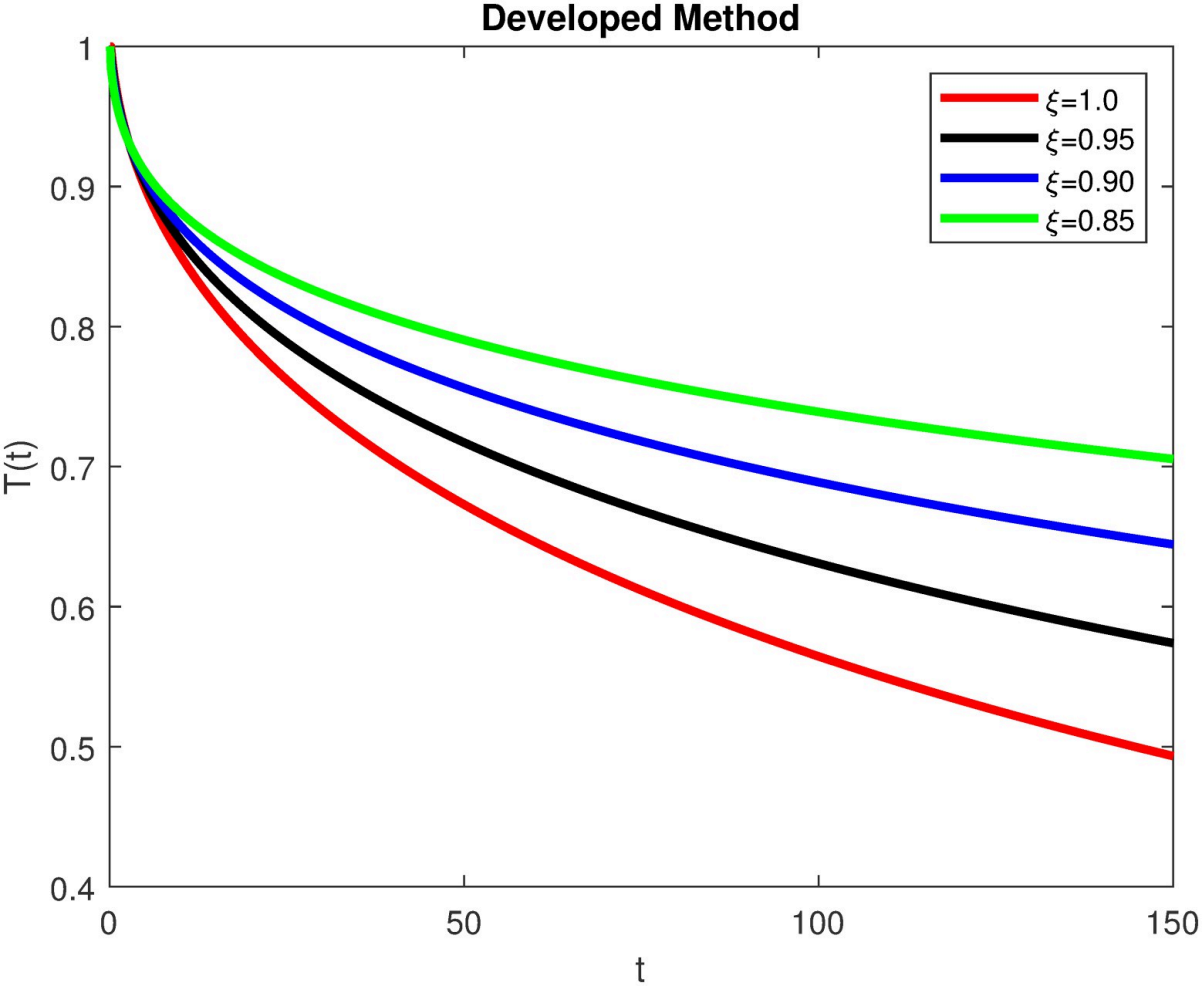
Tumor cells *T*(*t*) with dimension 0.5. The value of *T*(*t*) using fractal fractional operator with various fractional values at 0.5 dimension.

**Fig 3:**
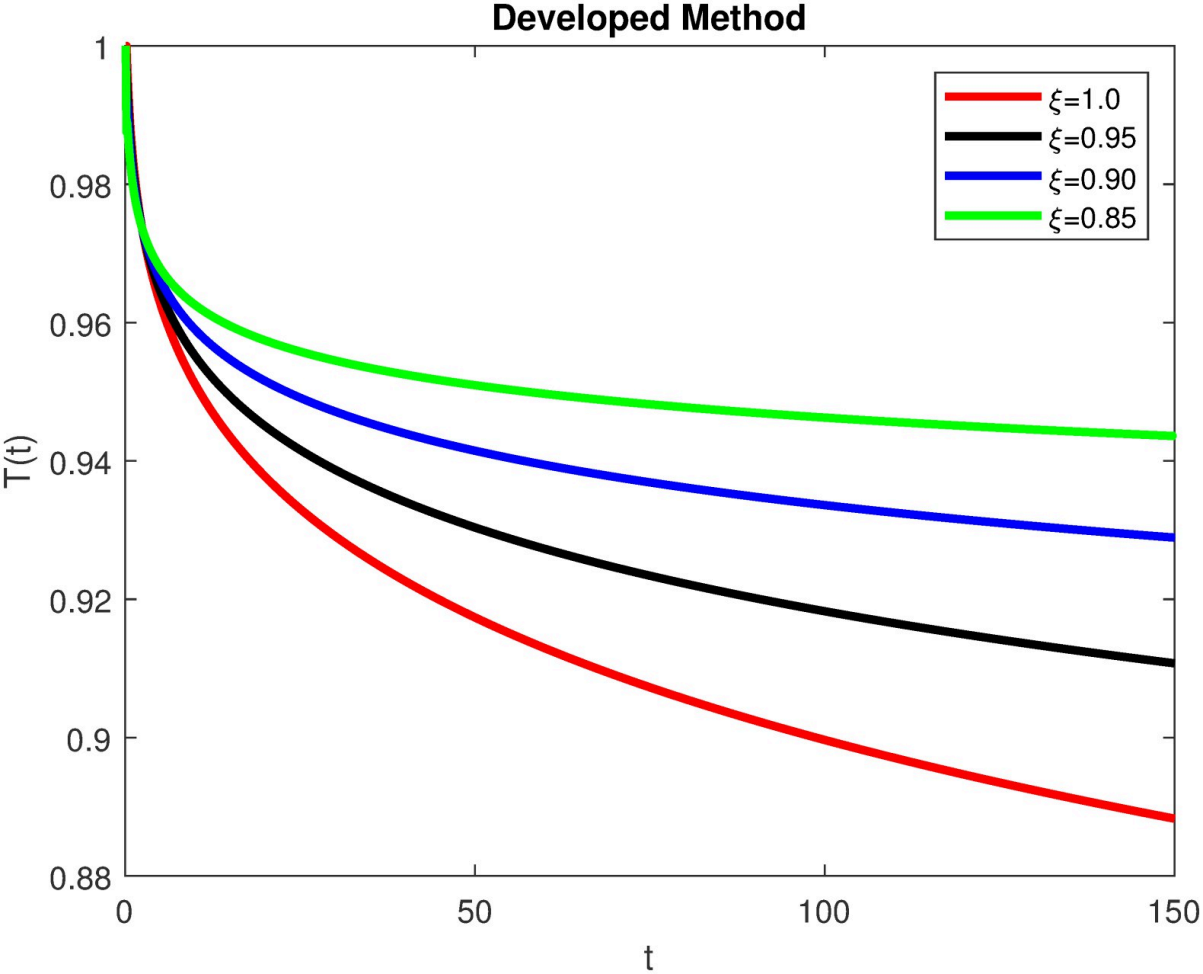
Tumor cells *T*(*t*) with dimension 0.2. The value of *T*(*t*) using fractal fractional operator with various fractional values at 0.2 dimension.

**Fig 4:**
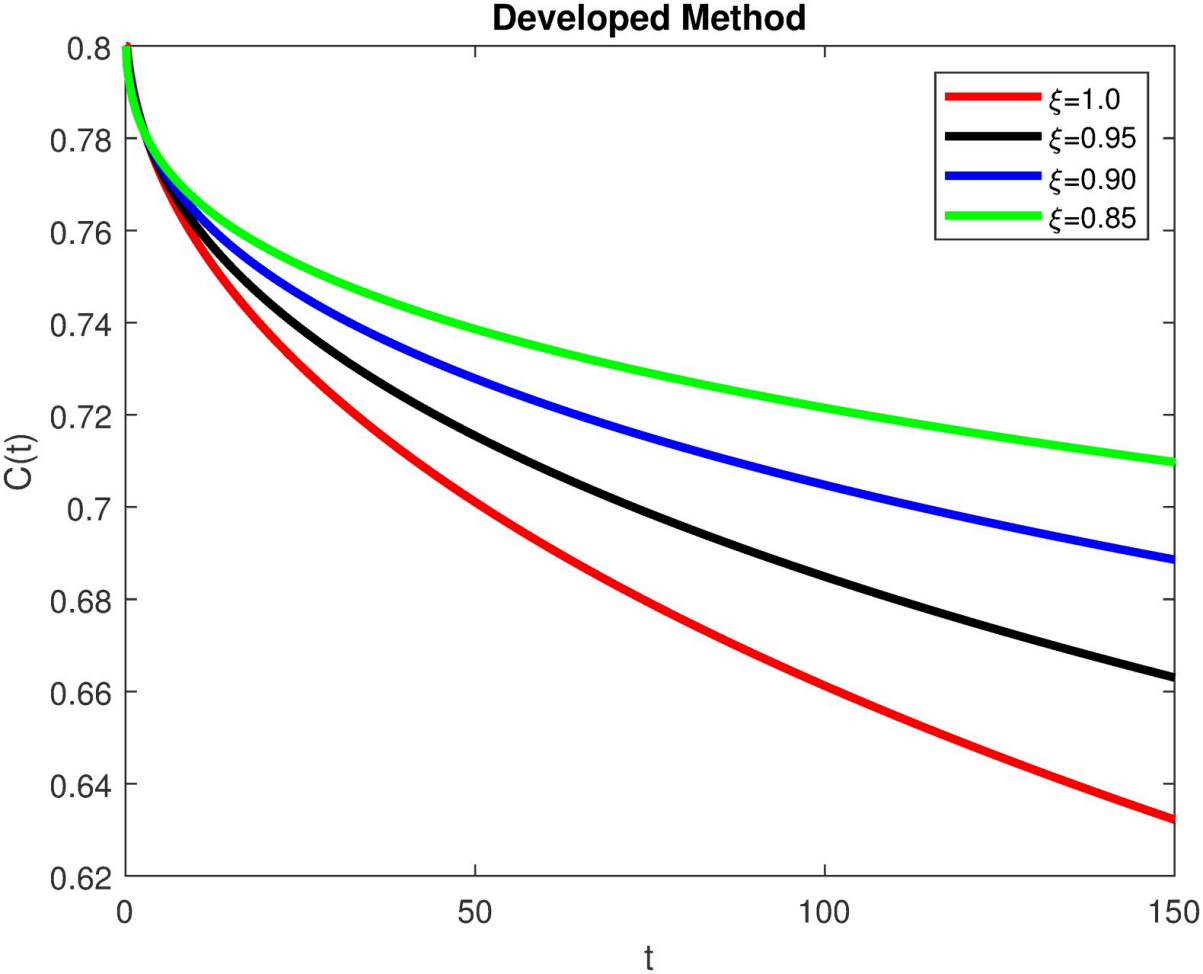
Cancer cells *C*(*t*) with dimension 0.5. The value of *C*(*t*) using fractal fractional operator with various fractional values at 0.5 dimension.

**Fig 5:**
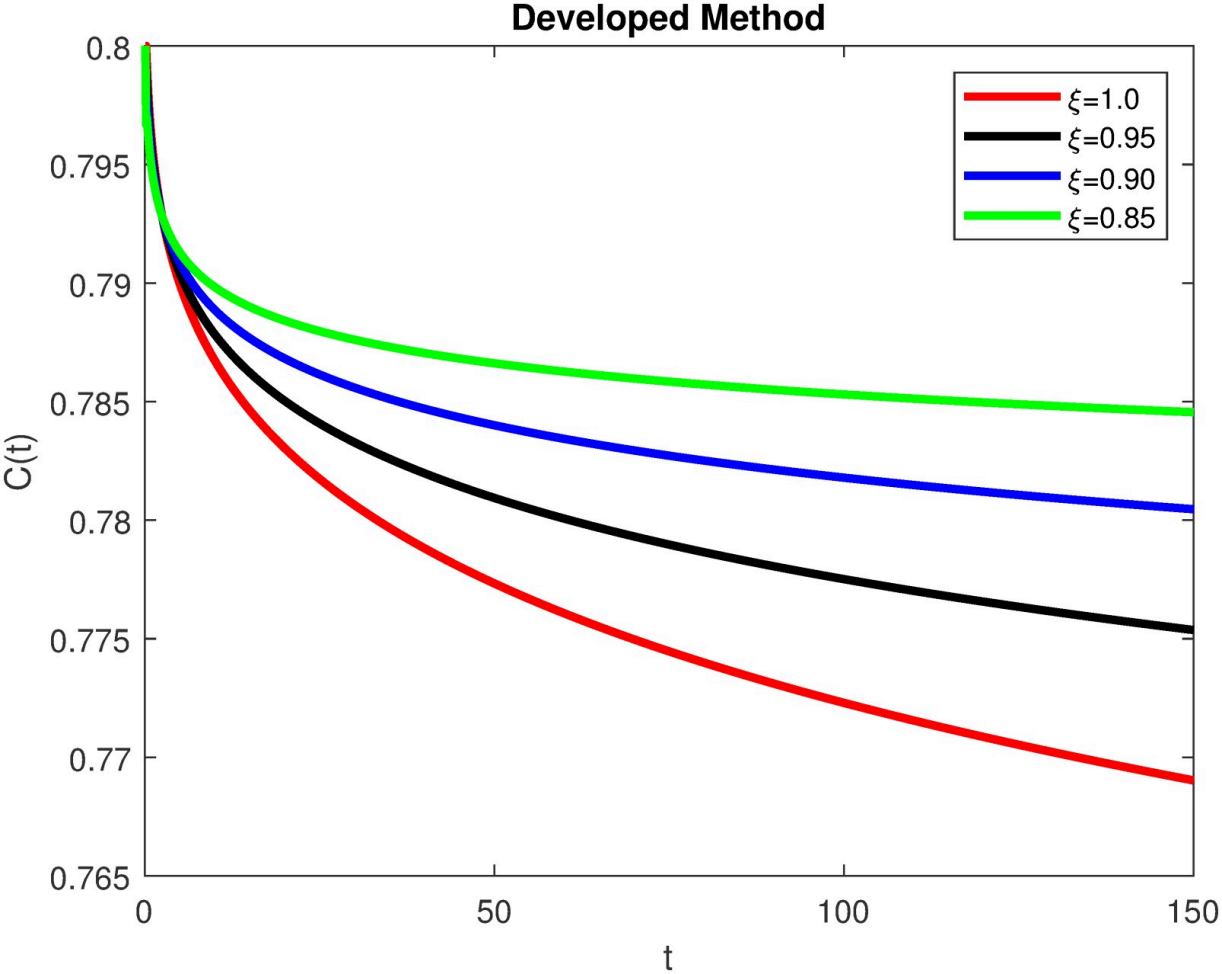
Cancer cells *C*(*t*) with dimension 0.2. The value of *C*(*t*) using fractal fractional operator with various fractional values at 0.2 dimension.

**Fig 6:**
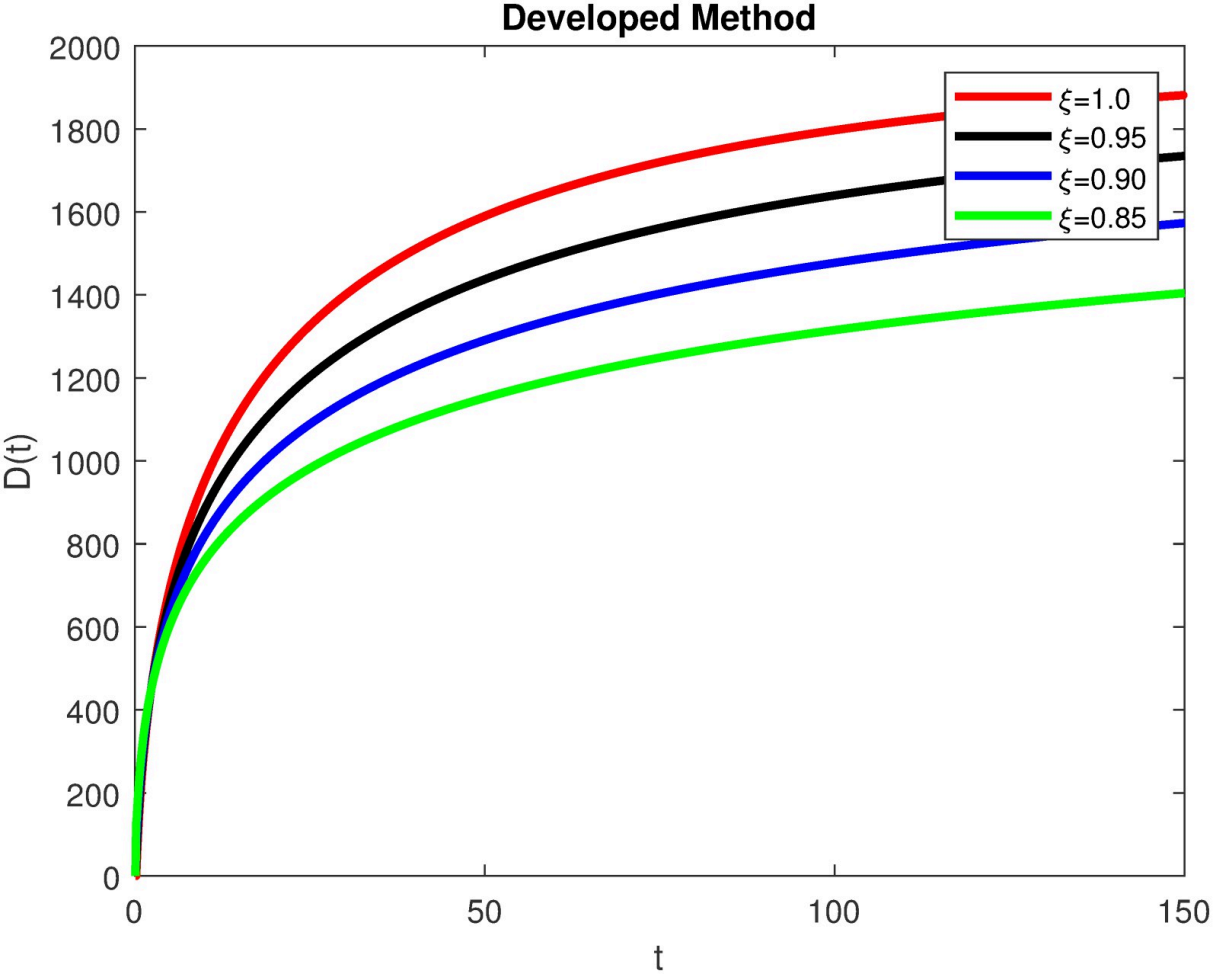
Dendritic cells *D*(*t*) with dimension 0.5. The value of *D*(*t*) using fractal fractional operator with various fractional values at 0.5 dimension.

**Fig 7:**
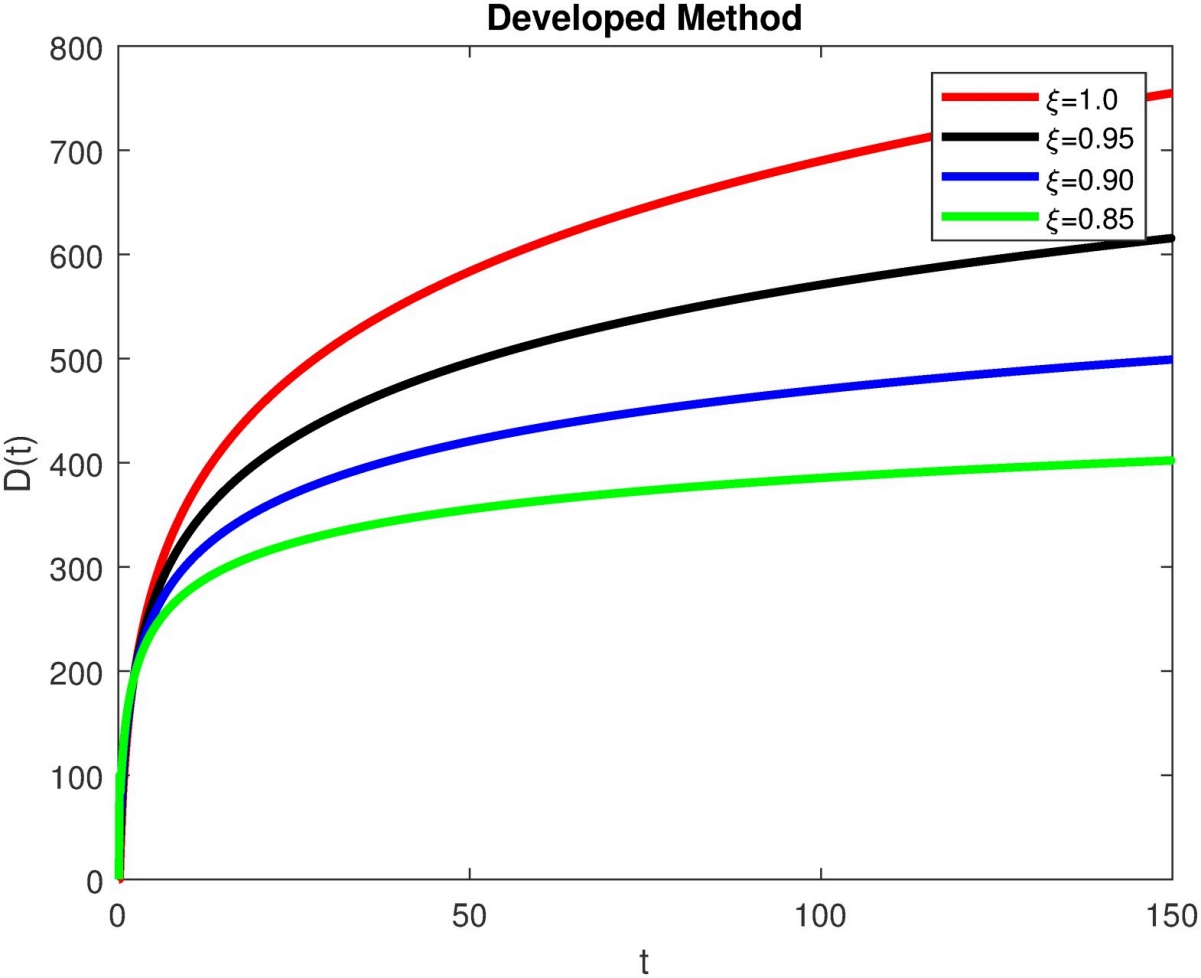
Dendritic cells *D*(*t*) with dimension 0.2. The value of *D*(*t*) using fractal fractional operator with various fractional values at 0.2 dimension.

**Fig 8:**
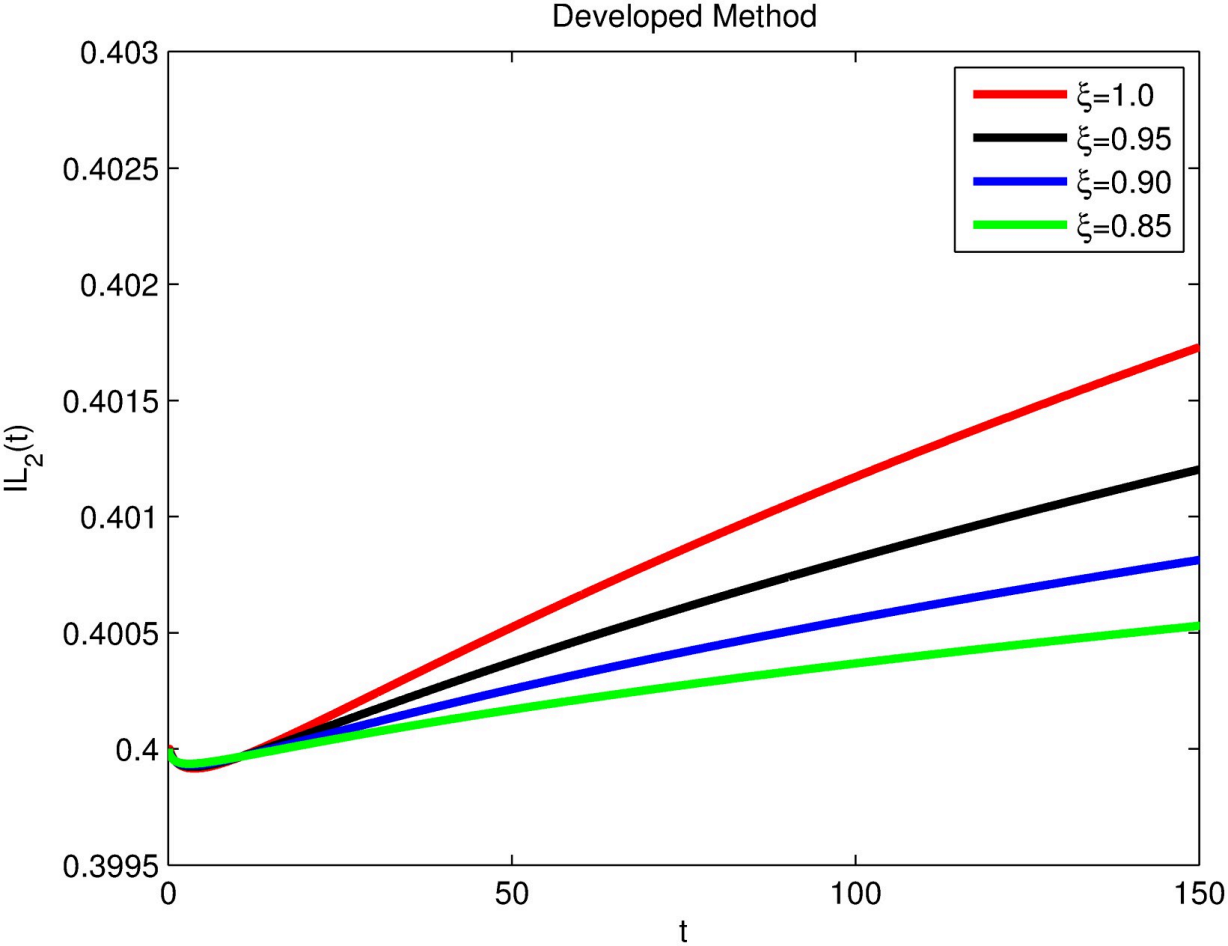
Cytokine *IL*_2_(*t*) with dimension 0.5. The value of *IL*_2_(*t*) using fractal fractional operator with various fractional values at 0.5 dimension.

**Fig 9:**
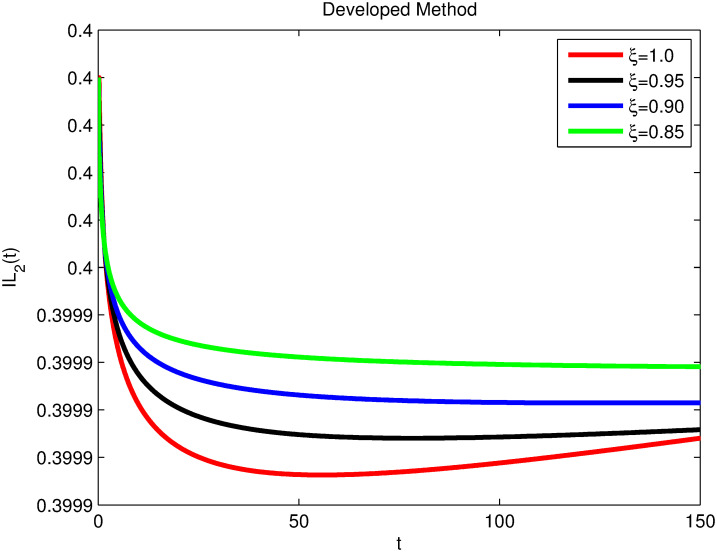
Cytokine *IL*_2_(*t*) with dimension 0.2. The value of *IL*_2_(*t*) using fractal fractional operator with various fractional values at 0.2 dimension.

**Fig 10:**
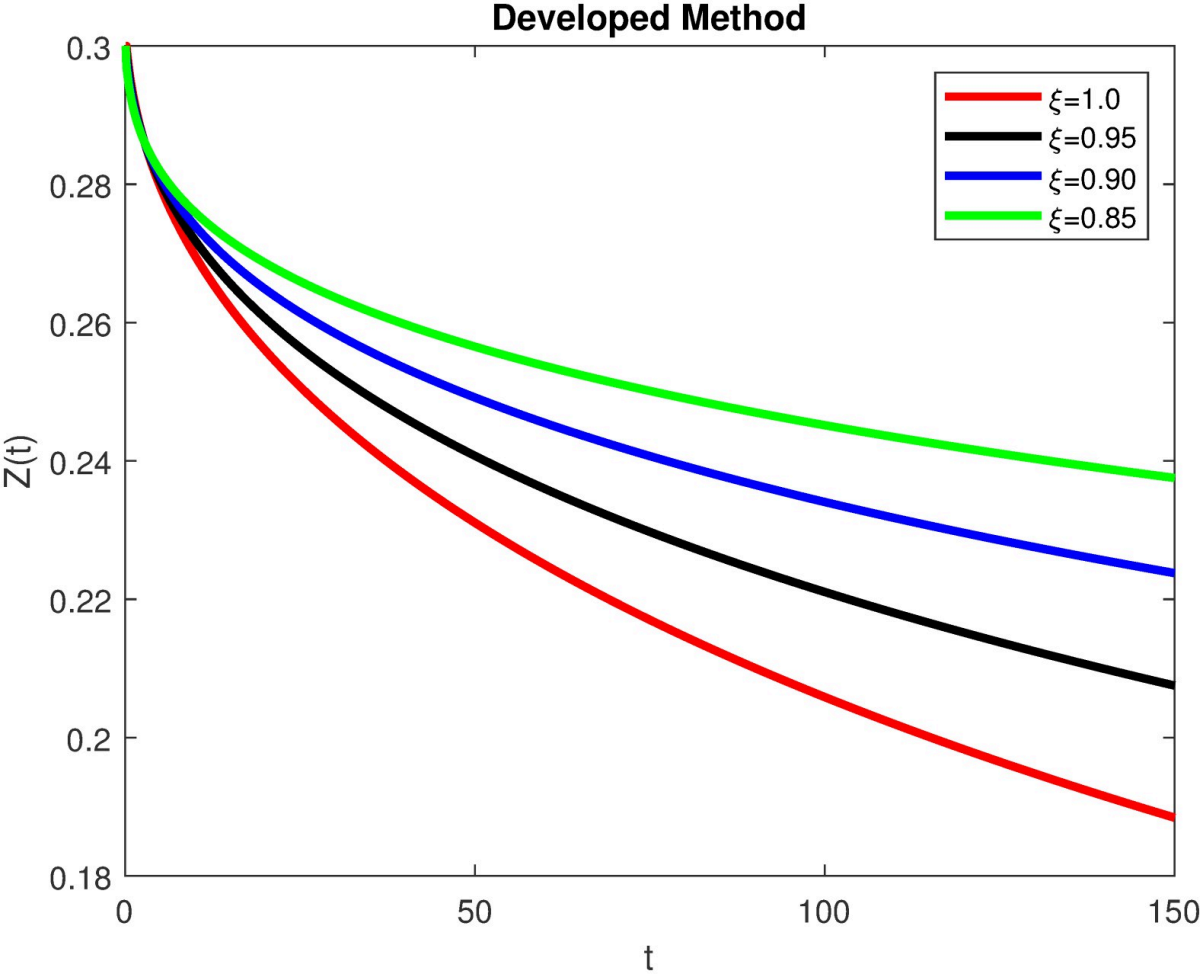
Anti-PD-L1 inhibitor *Z*(*t*) with dimension 0.5. The value of *Z*(*t*) using fractal fractional operator with various fractional values at 0.5 dimension.

**Fig 11:**
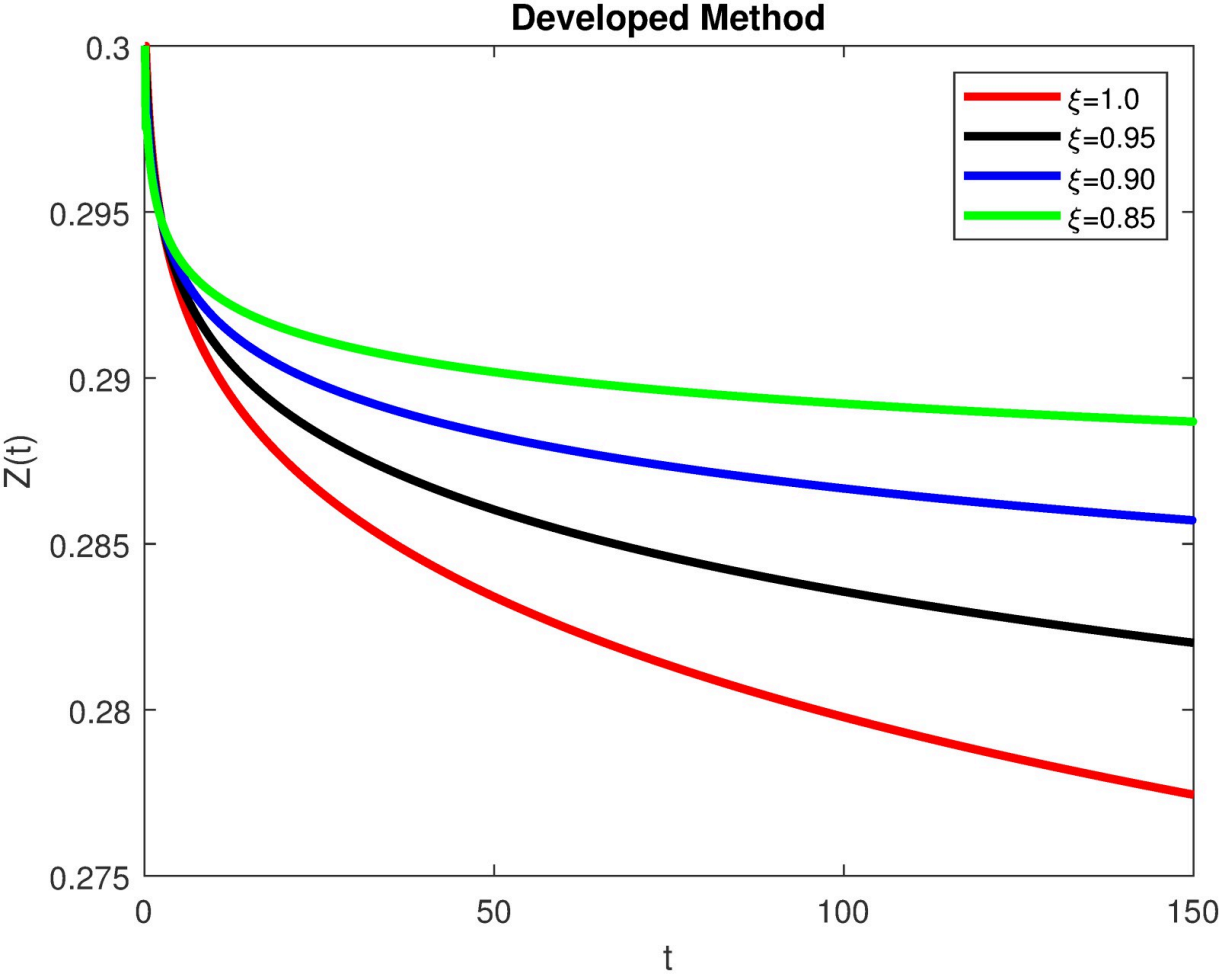
Anti-PD-L1 inhibitor *Z*(*t*) with dimension 0.2. The value of *Z*(*t*) using fractal fractional operator with various fractional values at 0.2 dimension.

The advance technique is used to obtain theocratical outcomes and investigate their effectiveness. The newly developed system TCD*IL*_2_Z is analyzed through simulation. We have achieved interesting findings through applying non-integer parametric values in lung cancer model. In the Figs 2–11, the individuals *T*(*t*), *C*(*t*), *D*(*t*), *IL*_2_(*t*) and *Z*(*t*) gives us the reliable solutions if we decrease fractional values. MATLAB code is used to determine the approximate solutions in simulations form for lung cancer model. The initial conditions used in newly developed model are *T*(0) = 1.0, *C*(0) = 0.8, *D*(0) = 0.3, *IL*_2_(0) = 0.4, and *Z*(0) = 0.3. The parameter values used in the developed system are *α* = 0.0514, *β* = 0.00000000102, *γ* = 0.1, *ϕ* = 0.0000001, *κ* = 0.02, *μ* = 480, *ρ* = 0.00000001 and *ω* = 0.24 which are taken from [34], where λ = 0.0000002, *d* = 0.0003 and *a* = 0.04 are assumed in the feasible range. Figs 2 and 3 represents the dynamics of Tumor cell *T*, Figs 4 and 5 represents the dynamics of Cancer cell *C*, Figs 8 and 9 represents the dynamics of cytokine *IL*_2_ and Figs 10 and 11 represents the dynamics of anti-PD-L1 inhibitor *Z* respectively in which all the compartments decreases sharply, and after certain time all compartments approaches to its stable position using different dimensions. Figs 6 and 7 represents the dynamics for dendritic cells *D* in which the individuals increases sharply, and after certain time all compartments approaches to its stable position using different dimensions. It is observed that cancer cells decline sharply due to *IL*_2_ cytokine and anti-PD-L1 inhibitor, can be seen in Figs 4, 5, 8–11 using different dimensions. It is observed that results are similar using dimension either 0.2 or 0.5 with minor effects, but it provide more appropriate results by decreasing dimensions and can be seen in Figs 10 and 11. It is also observed that *IL*_2_ and anti-PD-L1 cells help to increase the immune system of CD4+T and CD8+T lymphocytes production and decrease the cancer cells. The anti-PD-L1 also helps to create cells which are destroyed by cancer cells in the body as well as help to reduce these cancer cells [40]. It predicts what should happen in future by this research and how we shall be able to reduce the number of cancer unit spread in the body more efficiently. FFM (fractal fractional with Mittag-Leffler kernel)approach gives better results for all sub-compartment at different fractional derivatives, if we compare it with classical derivative. Also, it is stated that the solutions for all compartments are more trustworthy and accurate when fractional values are reduced as well as dimensions are reduced.

## 8 Conclusion

In this article, fractional order TCD*IL*_2_Z model for lung cancer is formulated by introducing *IL*_2_ cytokine and anti-PD-L1 inhibitor to boost up the immune for low immune individuals. We demonstrate advices to control the spread of disease by introducing the anti-cancer cell’s which improves the immune system of the individuals, so that the environment become disease free. The dangerous lung cancer disease is investigated with detection and treatment measures to examine the real impact of lung cancer in the world wide. For this purpose, the developed system is investigated quantitatively as well as qualitatively to verify its stable position for a continuous dynamical system. We also verify that the fractional order lung cancer disease model has bounded and unique solutions. We examine the impact of global measures to control the spread of the lung cancer disease as well as verify its existence. It is observed that the cancer infected individuals reduces due to *IL*_2_ and anti-PD-L1 inhibitor measures for low immune individuals. The Fractal-Fractional Operator (FFO) is used for continuously monitoring the spread of the disease using different fractional values as well as reliable and realistic findings. In fractal-fractional operators, fractal represents the dimensions of the spread of the disease and fractional represents the fractional ordered derivative operator which provide the real behavior of spread as well as control of lung cancer with different dimensions and continuous monitoring respectively and can be observed in simulation. We conduct numerical simulation with the help of MATLAB to see it’s real behavior of lung cancer disease to control the disease in the community after introducing *IL*_2_ cytokine and anti-PD-L1 inhibitor measures. Also the predictions can be made on the basis of our justified outcomes for future investigations which will be helpful to understand the behavior and outbreak of lung cancer disease spread in the environment as well as in early detection process.
